# Yield of tumor samples with a large guide-sheath in endobronchial ultrasound transbronchial biopsy for non-small cell lung cancer: A prospective study

**DOI:** 10.1371/journal.pone.0259236

**Published:** 2021-10-29

**Authors:** Naoko Katsurada, Motoko Tachihara, Naoe Jimbo, Masatsugu Yamamoto, Junya Yoshioka, Chihiro Mimura, Hiroki Satoh, Koichi Furukawa, Takehiro Otoshi, Tatsunori Kiriu, Yuichiro Yasuda, Tomonori Tanaka, Tatsuya Nagano, Yoshihiro Nishimura

**Affiliations:** 1 Division of Respiratory Medicine, Department of Internal Medicine, Kobe University Graduate School of Medicine, Kobe, Japan; 2 Department of Diagnostic Pathology, Kobe University Graduate School of Medicine, Kobe, Japan; PLOS Climate, UNITED KINGDOM

## Abstract

**Background:**

Adequate tumor tissue is required to make the best treatment choice for non-small cell lung cancer (NSCLC). Transbronchial biopsy (TBB) by endobronchial ultrasonography with a guide sheath (EBUS-GS) is useful to diagnose peripheral lung lesions. The data of tumor cell numbers obtained by two different sizes of GSs is limited. We conducted this study to investigate the utility of a large GS kit to obtain many tumor cells in patients with NSCLC.

**Methods:**

Patients with a peripheral lung lesion and suspected of NSCLC were prospectively enrolled. They underwent TBB with a 5.9-mm diameter bronchoscope with a large GS. When the lesion was invisible in EBUS, we changed to a thinner bronchoscope and TBB was performed with a small GS. We compared the tumor cell number prospectively obtained with a large GS (prospective large GS group) and those previously obtained with a small GS (small GS cohort). The primary endpoint was the tumor cell number per sample, and we assessed characteristics of lesions that could be obtained by TBB with large GS.

**Results:**

Biopsy with large GS was performed in 55 of 87 patients (63.2%), and 37 were diagnosed with NSCLC based on histological samples. The number of tumor cells per sample was not different between two groups (658±553 vs. 532±526, estimated difference between two groups with 95% confidence interval (CI); 125 (-125–376), p = 0.32). The sample size of the large GS group was significantly larger than that of the small GS cohort (1.75 mm^2^ vs. 0.83 mm^2^, estimated difference with 95% CI; 0.92 (0.60–1.23) mm^2^, p = 0.00000019). The lesion involving a third or less bronchus generation was predictive factors using large GS.

**Conclusions:**

The sample size obtained with large GS was significantly larger compared to that obtained with small GS, but there was no significant difference in tumor cell number. The 5.9-mm diameter bronchoscope with large GS can be used for lesions involving a third or less bronchus generation.

## Introduction

Worldwide, lung cancer is the leading cause of cancer-related deaths [1]. In recent years, targeted therapies matching with genomic alterations and immunotherapy have dramatically improved the survival of patients with non-small cell lung cancer (NSCLC) [2,3]. Molecular analysis targeting multiple genes and the evaluation of the programmed death ligand 1 (PD-L1) expression in tissue samples are essential to select optimal therapies for individual patients with NSCLC. The proportion of PD-L1 expression predicts the efficacy of an immune-checkpoint inhibitor [4]. Biopsy samples should contain at least 100 viable tumor cells on a sample for assessment of tumor proportion score to evaluate PD-L1 expression [5]. Recently, we have been able to simultaneously detect multiple genomic mutations by gene panel testing using next generation-sequencing (NGS) technology. An adequate tumor sample is required for successful gene panel testing. For example, to obtain the recommended 10-ng DNA for the Ion AmpliSeq^TM^ Cancer Hotspot Panel (Thermo Fisher Scientific, San Francisco, CA, USA), approximately 2000 tumor cells are required [6]. The proportion of tumor cells is also an important factor for molecular analysis [6]. Therefore, an adequate number of cells is required to determine the best treatment option for every individual patient.

Transbronchial biopsy (TBB) by endobronchial ultrasonography with a guide sheath (EBUS-GS) using virtual bronchoscopic navigation (VBN) has been recognized as a useful method to diagnose suspected lung cancer lesions. The reported diagnostic yield is 60–80% [7]. In our clinical practice, we can choose two different sizes of available GS kits for EBUS-GS—large and small—and a bronchoscope with a suitable working channel diameter for the GS kit (Table 1). TBB with a large GS kit could obtain larger specimens than that with a small GS kit. On the other hand, TBB with small GS using the thinner bronchoscope may have the advantage of reaching the more peripheral bronchi. A previous retrospective report investigating the diagnostic yield of EBUS-GS TBB for peripheral lung nodule (diameter, ≤30 mm) showed that the diagnostic yield was not different between the two sizes of GS, and that the choice of GS depended on the bronchoscopist’s decision [8]. In another prospective investigation, the diagnostic yield for peripheral lung nodule (diameter, ≤30 mm) using large GS for all cases was 74.4% [9]. These studies showed that the diagnostic yield of TBB with a large GS kit may be as similar as that with a small GS kit. However, the data related to cell numbers and sample size obtained by the two different GS kits are limited. To our knowledge, only one other study has prospectively investigated and compared the sample size and cell numbers between the two sizes of GS kits. They reported that tumor cell numbers obtained by TBB with large GS were larger than those obtained by small GS, where the selection of the GS depends on the bronchoscopist [10]. It is also unclear which lesion can be diagnosed by TBB with large GS, because the selection of the GS is usually decided by the bronchoscopist.

**Table 1:** Two different sizes of guide-sheaths and the suitable bronchoscope.

	Large Guide-sheath		Small Guide-sheath	
Suitable Bronchoscope	5.9-mm diameter bronchoscope		Thinner bronchoscope	
	1T260	1TQ290	P290	P260F
Bronchoscope diameter	5.9 mm		4.2 mm	4.0 mm
Working channel diameter	2.8 mm	3.0 mm	2.0 mm	
Guide-sheath	SG-201C, SG-401C		SG-200C, SG-400C	
Guide-sheath diameter	2.5 mm		1.9 mm	
Forceps	FB-231D		FB-233D	
Forceps diameter	1.9 mm		1.5 mm	

All bronchoscopes and related equipment were manufactured by Olympus (Tokyo, Japan).

We conducted this study to investigate the utility of a large GS kit to obtain many tumor cells in patients with NSCLC. We further assessed the characteristics of lesions that could be obtained by TBB with large GS. We used a large GS kit for all prospectively enrolled patients and compared the tumor cell numbers between samples obtained by the large GS vs. those previously obtained by a small GS. We hypothesized that samples obtained by TBB with a large GS kit contain more tumor cells than those obtained by TBB with a small GS kit.

## Material and methods

### Study design

This prospective study was conducted to evaluate the utility of a large GS kit to obtain more tumor cells in patients with NSCLC. We prospectively enrolled patients scheduled to undergo EBUS-GS TBB for peripheral lung lesions suspected of being NSCLC and performed EBUS-GS TBB using large GS kit. Furthermore, we enrolled consecutive patients with NSCLC in whom tissue samples were previously obtained by EBUS-GS TBB using a small GS kit. We compared tumor cell numbers of samples obtained from patients who were histologically diagnosed with NSCLC by TBB with large GS (prospective large GS group) and those obtained from patients who were previously diagnosed with NSCLC by TBB with small GS (small GS cohort).

### Patients

The eligibility criteria were age ≥20 years and patients with undiagnosed peripheral lung lesion, suspected of being NSCLC on computed tomography (CT). The physicians referred this study to the participant when they decided that bronchoscopy was necessary for a lesion suggestive of NSCLC. Written informed consent was obtained from all patients. The peripheral pulmonary lesion was defined as not visible by bronchoscopy. Exclusion criteria were patients with visible lesion on bronchoscopy, those who underwent re-biopsy after treatment of lung cancer, those who had severe comorbidities such as severe cardiac diseases or insufficient pulmonary function, patients who could not discontinue antiplatelet or anticoagulant therapy, pregnant woman, and patients judged to be unsuited for this study by their physicians. We also enrolled consecutive NSCLC patients who previously underwent EBUS-GS TBB using a small GS kit in our institution. We applied the opt-out method to obtain consent on retrospectively enrolled patients. This study was approved by the ethics committee of Kobe University (300016) on July 24, 2018. And it was conducted in accordance with the Helsinki declaration. This study is registered in the University Medical Hospital Information Network in Japan (UMIN 000032599,　https://upload.umin.ac.jp/cgi-open-bin/ctr/ctr_view.cgi?recptno=R000036780) and the date of registration was August 22, 2018. Participants were recruited from August 23, 2018 to March 31, 2020. We prospectively recruited consecutive patients who were planning to be performed biopsy by EBUS-GS TBB in clinical practice in our institution, so sample of this study can be considered of a larger population.

### CT evaluation of the lesion

Two pulmonologists evaluated the lesion size, location, presence, or absence of bronchus sign [11]. The location of the lesion was classified as central, intermediate, or peripheral based on the distance from the hilum on CT. Lesions located within the inner third area were considered central, those located within the middle third area were intermediate, and those located within the outer third area were considered peripheral [12]. The lesion characteristics on CT scan were classified as solid, part-solid ground-glass opacity (GGO), and pure GGO. Subsegmental bronchi were regarded as third-generation bronchi, and the number of bronchi was calculated by adding the number of further branchings.

### Bronchoscopy procedure

All patients underwent CT scanning (slice width: 1.0 mm) before bronchoscopy. The bronchial path to the lesion was planned using a chest CT or virtual bronchoscopic navigation software (Bf-NAVI^®^; Cybernet Systems, Tokyo, Japan) that automatically created a virtual bronchial image of the target lesion [13]. Bronchoscopy was performed through the oral route under local anesthesia with conscious sedation. All bronchoscopies and devices were manufactured by Olympus, Tokyo, Japan. The bronchoscope, BF 1T260 (5.9 mm outer diameter, 2.8 mm working channel diameter) or 1TQ290 (5.9 mm outer diameter, 3.0 mm working channel diameter), was advanced to the target lesion through the planned bronchial route. A curette-type inductor (CC-6DR-1) was permitted to use to insert the GS into the appropriate bronchial branch. When the bronchoscope reached near the target lesion, the large GS (SG-201C or SG-401C, 2.5 mm external diameter) was inserted with UM-S20-20R radial EBUS probe through the working channel. If the target lesion could be visualized under EBUS, the EBUS probe was withdrawn and the forceps (FB-231D, diameter: 1.9 mm) were inserted through the GS. Specimens were obtained from the same lesion using forceps introduced into the GS as well as by brushing twice. After at least five specimens were obtained, the GS was aspirated with 20 mL of negative pressure for 20 seconds to collect cells and withdraw the GS [14]. If the target lesion was not visible by EBUS, the bronchoscope was changed to the thinner type—BF P290 (4.2 mm outer diameter, 2.0 mm working channel diameter) or BF P260F (4.0 mm outer diameter, 2.0 mm working channel diameter)—and advanced to the target lesion. When the thinner bronchoscope reached the lesion, the small GS (SG-200C or SG-400C, 1.9 mm external diameter) was inserted with UM-S20-17S radial EBUS probe through the working channel. Similar to the procedure with large GS TBB, specimens were obtained using forceps (FB-233D, diameter: 1.5 mm) and a brush introduced into the GS. Additional conventional TBB using biopsy forceps (FB-231D) and transbronchial needle aspiration using aspiration needle (MAJ-64) were permitted. X-ray fluoroscopy was intermittently used to guide the EBUS probe and biopsy devices to the target lesion and confirm movement of the devices during sample collection. We recorded the number of branches that can be observed under bronchoscopy and endobronchial ultrasonography images.

### Pathological evaluation

Pathological diagnosis was confirmed by a histopathologist based on hematoxylin-eosin (HE) cell staining. After a confirmed diagnosis of NSCLC, we evaluated the number of tumor cells and sample size of the first consecutive five specimens in the large GS group and small GS cohort. We scanned the HE-stained slides with a scanner (Nano zoomer® 2.0RS, HAMAMATSU, Japan). A pathologist and a cytologist, blinded to the clinical details, manually counted the number of tumor cells and evaluated the proportion of tumor cells in all nuclear cells using imaging software (NDP scan^®^ 2.5, HAMAMATSU, Japan), and average number and proportion assessed by the two evaluators. Only viable tumor cells were counted, and those that were difficult to differentiate from non-tumor cells, such as inflammatory cells, or those with strong degeneration or necrosis were excluded. The sample size was measured by the cumulative area of individual specimens using imaging software (cellSens^®^ standard, Olympus, Japan). PD-L1 was evaluated using the samples containing the largest number of tumor cells obtained by GS-TBB.

### Endpoints

The primary endpoint was comparison of the mean tumor cell number per sample of first five consecutive samples between the prospective large GS group and the small GS cohort. We set the tumor cell numbers as the surrogate endpoint to assess evaluability of molecular analysis because the gene panel testing could not be routinely used in clinical practice in most of the study period. The secondary endpoint was to evaluate the number of tumor cells containing the largest number of tumor cells among first five consecutive samples, sample size, proportion of tumor cells in nucleated cells, and the success rate of PD-L1 testing between prospective large GS group and small GS cohort. In the prospectively enrolled patients, we evaluated factors associated with change to a thinner bronchoscope in order to clarify the characteristics of lesions that could be obtained by TBB with large GS. We evaluated the tumor diameter, tumor location, tumor characteristics, CT bronchus sign, bronchus generation constructed by VB images, and the bronchus generation visible by bronchoscopy between lesions that did not require to change to the thinner bronchoscope (not changed group) and those that needed this change (changed group). Last, we evaluated the safety of prospectively performed bronchoscopy.

### Sample size and statistical analyses

In a pilot study, the average tumor cell counts in two patients who underwent TBB with large GS were 1410 and 302. Further, in three cases that underwent EBUS-GS TBB using small GS, the average tumor cell counts were 192, 407, and 675. We transformed the pilot data to the logarithmic scale, obtaining the two means as 6.4808 and 5.9270 (difference 0.5538) respectively and standard deviation common to the two groups was 0.8139. The sample size was calculated assuming that the mean difference between the two groups was 0.5538, with an alpha level set at 0.05 (two-sided) and detection power of 80%. The minimum sample size was calculated as 34 for each group (prospective large GS group and small GS cohort), and was set at 80 for the prospective large GS group assuming that cases that required to change the thinner bronchoscope, whose pathological diagnosis other than NSCLC and deviation. The chi-square or Fisher’s exact tests were used for qualitative data; Student’s *t*-test and Wilcoxon Mann–Whitney test were used for quantitative data. Clinically relevant factors associated with diagnostic yield were selected for the multivariate logistic regression analysis model to evaluate which lesion was suitable for TBB with large GS. All statistical analyses were performed using EZR version 1.38 (Saitama Medical Center, Jichi Medical University, Saitama, Japan), a graphical user interface for R (version 3.3.2; R Foundation for Statistical Computing, Vienna, Austria) [15].

### Study follow-up

If the lesion was not diagnosed, patients were recommended to undergo another diagnostic procedure such as CT-guided transthoracic needle aspiration biopsy (CTNB), repeated bronchoscopy, or surgery. In the event that the patient did not require further diagnostic procedures, the lesion was followed-up for 2 years. The final diagnosis was based on pathological evaluation or clinical follow-up.

## Results

### Consort flow chart and diagnosis

**Fig 1:**
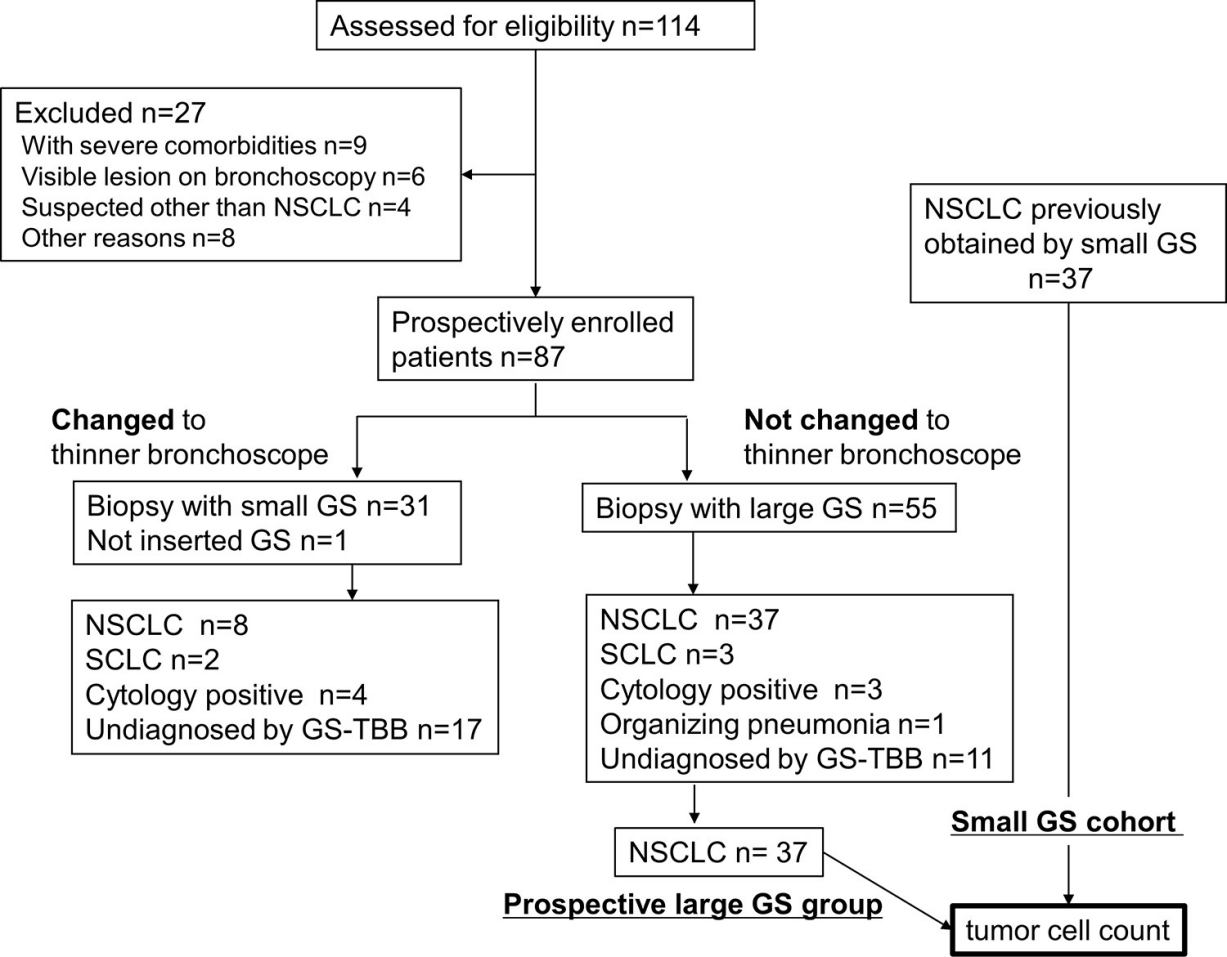
Consort flow chart. GS; guide sheath, NSCLC; non-small cell lung cancer, SCLC; small cell lung cancer, TBB; transbronchial biopsy.

Fig 1 shows the Consort flow chart. From August 2018 to March 2020, 87 patients were prospectively enrolled. And we retrospectively enrolled patients in whom tumor samples were obtained by EBUS-GS TBB using a small GS kit from April to December 2017. Table 2 shows the demographic and clinical characteristics of prospectively enrolled patients. All eligible patients underwent bronchoscopy; 32/87 (36.8%) patients required to change a bronchoscope to a thinner one. Further, 55/87 (63.2%) patients did not need a change of bronchoscope and underwent biopsy with large GS. Among these 55 patients, 37 were diagnosed with NSCLC and three were diagnosed with SCLC based on histological biopsy samples, three were diagnosed with NSCLC by cytology, one was diagnosed with organizing pneumonia, and the remaining 11 patients were not diagnosed by GS-TBB. The diagnostic yield of large GS was 50.6% (44/87). We evaluated tumor cell numbers of samples obtained from 37 patients who were histologically diagnosed with NSCLC by TBB with large GS. Among the 32 patients who required a change to the thinner bronchoscope, the target lesion became visible under ultrasonography in 71% (22/31); the small GS could not be inserted in one patient because of an adverse event during the procedure. Among the 31 patients, 10 were diagnosed with lung cancer based on histological biopsy samples, four were diagnosed with lung cancer by cytology, and 17 patients were not diagnosed by GS-TBB. Therefore, 58 of 87 (66.7%) patients who were prospectively enrolled were diagnosed by bronchoscopy. Among 28 patients undiagnosed by bronchoscopy, 18 were diagnosed with lung cancer, one was diagnosed with malignant mesothelioma, and two were diagnosed with inflammatory changes by CTNB or surgery. Five patients were followed-up, and two were lost to follow-up. We evaluated tumor cell numbers of samples obtained from 37 patients who were histologically diagnosed with NSCLC by TBB with large GS (prospective large GS group) and those previously obtained from 37 patients who were diagnosed with NSCLC by TBB with small GS (small GS cohort).

**Table 2:** Characteristics of the 87 patients who were prospectively enrolled.

	n (%)
Age (median; range)	72 (54–88)
Sex (male)	71 (81.6)
Smoking status	
Current/former	71 (81.6)
Never	16 (18.4)
Lesion size (mm, median; range)	21.5 (9.0–73.0)
Lobar location	
Right upper lobe	25 (28.7)
Right middle lobe	6 (6.9)
Right lower lobe	20 (23.0)
Left upper lobe	23 (26.4)
Left lower lobe	13 (14.9)
Lesion location from hilum	
Central	8 (9.2)
Intermediate	15 (17.2)
Peripheral	64 (73.6)
Bronchus sign	
Present	84 (96.6)
Absent	3 (3.4)
Characteristics	
Solid	80 (92.0)
Part solid GGO	7 (8.0)
Bronchus generation on VBN (mean±SD, n = 86)	4.6±1.1

GGO; ground-glass opacity, VBN; virtual bronchoscopic navigation.

### Tumor cell number

**Fig 2:**
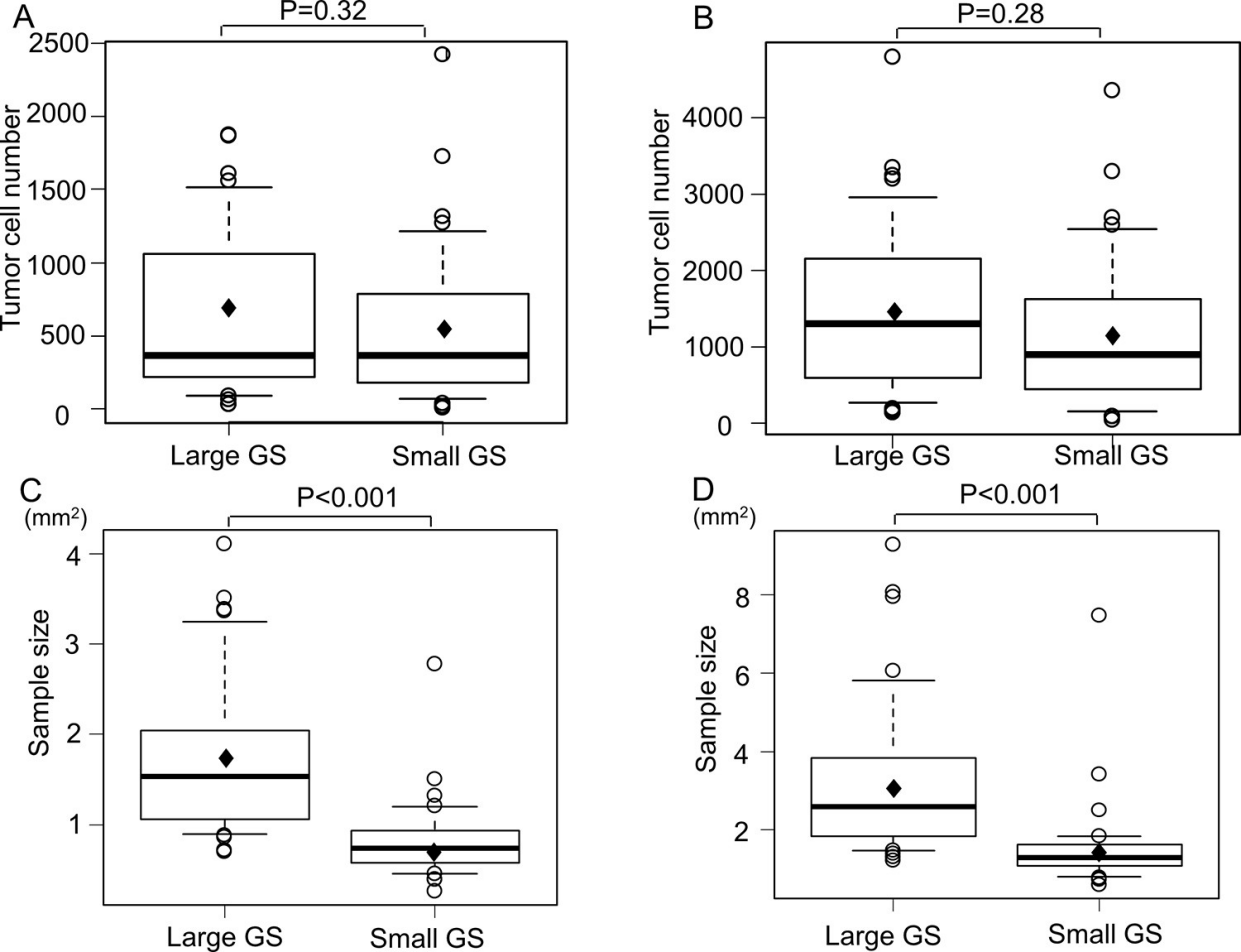
Tumor cell counts and sample sizes between prospective large GS group and small GS cohort. A) The comparison of the average number of tumor cells from five samples between two groups. B) The comparison of the cell counts of the sample containing the largest number of tumor cells among five samples between two groups. C) The comparison of the average sample size of five samples between two groups. D) The comparison of the largest sample size of five samples between two groups. GS; guide sheath. ◆; mean value.

Table 3 shows the pathological subtypes, cell counts, and sample sizes between the prospective large GS group and the small GS cohort. Fig 2 shows the comparison between the number of tumor cells and sample size between the two groups. The frequency of pathological subtypes, CT and EBUS findings, and the proportion of tumor cells in nucleated cells were not different between these two groups. The number of tumor cells per sample in the large GS group was not different from that in the small GS cohort. (large GS; 658±553 vs. small GS; 532±526, estimated difference between two groups with 95% confidence interval (CI); 125 (-125–376), p = 0.32). The mean cell counts of the sample containing the largest number of tumor cells among five consecutive samples (the maximum tumor cell number) in the large GS group was not different from that of the small GS group (1436±1103 vs. 1170±994, estimated difference with 95% CI; 266 (-221–752), p = 0.28). There were slightly more patients in the large GS group with maximum number of tumor cells (>2000) than in the small GS group, but, again, this difference was not statistically significant (29.7% vs. 18.9%, estimated difference with 95% CI; 10.8 (-8.6–30.2)%, p = 0.42). The mean sample size of the prospective large GS group was larger than that of the small GS cohort (1.75±0.87 mm^2^ vs. 0.83±0.42 mm^2^, estimated difference with 95% CI; 0.92 (0.60–1.23) mm^2^, p = 0.00000019). The largest sample size of five samples in the large GS group was also larger than that of the small GS cohort (3.22±2.02 mm^2^ vs 1.53±1.13 mm^2^, estimated difference with 95% CI; 1.69 (0.93–2.45) mm^2^, p = 0.0000333). The proportion of evaluable PD-L1 expression of the prospective large GS group tended to be more than that of the small GS cohort (100.0% vs. 89.7%, estimated difference with 95% CI; 10.3 (-0.7–21.4)% p = 0.08). The proportion of tumor cells in nucleated cells was not different between the two groups.

**Table 3:** Tumor cell counts and sample sizes between prospective large GS group and small GS cohort.

	Prospective large GS	Small GS cohort	p-value
	n = 37	n = 37	
Age (median; range)	72 (58–85)	71 (54–88)	0.72
Sex; male, n (%)	32 (86.5)	24 (64.9)	0.06
CT characteristic			
Solid	36 (97.3)	33 (89.2)	0.36
Part solid GGO	1 (2.7)	4 (10.8)	
EBUS findings			
Within	34 (91.9)	25 (94.6)	1.00
Adjacent to	3 (8.1)	2 (5.4)	
Tumor cell number per slide (mean±SD)	658±553	532±526	0.32
Maximum tumor cell number (mean±SD)	1436±1103	1170±994	0.28
Mean sample size (mm^2^, mean±SD)	1.75±0.87	0.83±0.42	<0.001
Largest sample size (mm^2^, mean±SD)	3.22±2.02	1.53±1.13	<0.001
Maximum proportion of tumor cells (%, median; range)	30 (5–80)	30 (1–90)	0.89
Median proportion of tumor cells (%, median; range)	10 (0–60)	10 (0–70)	0.97
Maximum tumor cell number			
<2000	26 (70.3)	30 (81.1)	0.42
≥2000	11 (29.7)	7 (18.9)	
PD-L1 evaluability	36/36 (100.0)	26/29 (89.7)	0.08

GS; guide sheath, PD-L1; programmed death ligand 1, GGO; ground-glass opacity, EBUS; endobronchial ultrasonography.

### Factors associated with change to a thinner bronchoscope

Table 4 shows the characteristics of lesions in patients who underwent biopsy with large GS (not changed group) and patients who required a change to the thinner bronchoscope (changed group) among those who prospectively underwent bronchoscopy. There was no difference in lobar location and lesion location from the hilum. In the not changed group of patients, lesion size was larger, presence of bronchus sign was more frequent, and lesser bronchus generation was noted toward the target lesion on VBN than those in changed group (4.40±1.17 vs. 4.93±0.84, estimated difference with 95% CI; 0.53 (0.059–1.00), p = 0.028). Logistic regression analysis indicated that the bronchus generation constructed by VBN was significantly associated with the change to the thinner bronchoscope. The mean bronchial generations accessed with the 5.9-mm diameter bronchoscope and the thinner bronchoscope were 3.26±0.8 and 4.13±0.8, respectively (estimated difference with 95% CI; 0.87 (0.53–1.21), p = 0.0000016). The mean bronchial generations constructed by VBN was 4.6±1.1. Of the 12 lesions involving a third or less bronchus generation, 11 (91.7%) were visible on EBUS using large GS, and 10 (83.3%) were diagnosed by GS-TBB with large GS. The diagnostic rates of TBB using large GS for the upper lobe and the other lobes were 42.9% (18 of 42) and 57.8% (26 of 45), respectively, and there was no significant difference (estimated difference with 95% CI; -14.9 (-35.7–5.9)% p = 0.24).

**Table 4:** Factors associated with change to a thinner bronchoscope.

	Changed n = 32	Not changed n = 55	p-value
Lesion size (mm, median; range)	21.5 (9.0–73.0)	31.0 (14.0–93.0)	0.01
Lobar location			
Right upper lobe	9 (28.1)	16 (29.1)	0.84
Right middle lobe	3 (9.4)	3 (5.5)	
Right lower lobe	6 (18.8)	14 (25.5)	
Left upper lobe	10 (31.2)	13 (23.6)	
Left lower lobe	4 (12.5)	9 (16.4)	
Lesion location from hilum			
Central	2 (6.2)	6 (10.9)	0.49
Intermediate	4 (12.5)	11 (20.0)	
Peripheral	26 (81.2)	38 (69.1)	
Bronchus sign			
Present	29 (90.6)	55 (100.0)	0.047
Absent	3 (9.4)	0 (0.0)	
Characteristics			
Solid	29 (90.6)	51 (92.7)	0.71
Part solid GGO	3 (9.4)	4 (7.3)	
Bronchus generation on VBN (mean±SD) n = 86	4.93±0.84	4.40±1.17	0.03
Multivariate analysis			
	HR *	95%CI	
Lesion size (mm)	0.98	0.95–1.01	0.22
Lobar location	1.09	0.79–1.50	0.64
Bronchus generation on VBN (mean±SD) n = 86	1.60	1.04–2.46	0.03

GGO; ground-glass opacity, VBN; virtual bronchoscopic navigation.

*Compared to not-changed group.

### Safety

There were two complications: one patient died due to acute aortic dissection during bronchoscopy and the other had lung abscess requiring hospitalization several days after bronchoscopy.

## Discussion

To our knowledge, this is the first prospective report to investigate the number of tumor cell samples obtained with large GS and those obtained by small GS in a clinical study setting to use large GS for all eligible patients in prospective groups. This study did not show that tumor cells obtained by large GS was more than those obtained by small GS although the size of samples obtained by large GS was significantly larger than that obtained by small GS. We established the primary endpoint as the average cell numbers per sample, and this study did not reach the primary endpoint. In the sample containing the largest number of tumor cells among five consecutive samples, cell numbers >2000 was slightly more frequent in the large GS group than the small GS cohort although there was not significant difference. In the clinical setting, we chose one sample containing the largest number of tumor cells for molecular analysis. Given that large specimens containing numerous tumor cells are generally essential for molecular testing, at the very least, one sample should contain enough cells. PD-L1 expression was evaluated in all patients in the large GS group, but 11% of cases were not evaluated in the small GS cohort. With these results, we believe TBB with large GS still have the potential for molecular analysis. In Japan, EBUS-GS TBB has been commonly used in many facilities, so large GS might be useful to obtain a large number of tumor cells in many institutions. We did not obtain a significantly large number of tumor cells with large GS, although the sample size of the large GS group was larger than that of the small GS group. Although the exact reason for this is unclear, it is likely that tissue other than tumor cells might be contained in samples obtained by TBB with large GS. In the clinical setting, we can perform microdissection to select the most appropriate tumor area for gene panel testing when the tumor cell number is not enough although the sample size is large. We think the molecular testing is required to investigate the utility of large GS and large forceps for TBB.

In this study, we have also clarified which lesion is suitable for using large GS: lesions that have less number of bronchial branches in the route constructed by VBN (<4th generation) are most likely suitable candidates for large GS use. The bronchus generation was significantly associated with the change to the thinner bronchoscope, this result suggests that lesions involving less bronchial branches is suitable for using large GS. The diagnostic rate of lesions involving a third or less bronchus generation biopsied with a large GS was 83.3%. We can use several different diameters of bronchoscopes and various instruments such as forceps, brush, and aspiration needle. The smaller-diameter bronchoscope can reach a further generation of the bronchus and can more correctly guide it to the target bronchus [16]. Bronchoscopes with smaller diameter have less diameter of the working channel which allows the use of smaller forceps. A previous study showed that the diagnostic yield of the ultrathin bronchoscope (outer diameter: 3.0 mm, working channel: 1.7 mm) was better than that of the thin bronchoscope (outer diameter: 4.0 mm, working channel: 2.0 mm) for small peripheral pulmonary lesions (diameter≤30 mm) [17]. They showed that the mean bronchial generations accessed with the ultrathin bronchoscope, thin bronchoscope, and VBN were 5.5, 4.4, and 5.1, respectively [17]. These findings were consistent with our study (the bronchial generation reached by the thinner bronchoscope was 4.1±0.8). Thinner bronchoscopes have an advantage in diagnostic yield, but samples obtained by small forceps are smaller than those obtained by large forceps. We need to gain a large number of tumor cells for personalized medicine in patients with NSCLC. The most important thing is to choose the best size of bronchoscope and forceps for the individual tumor to obtain a larger amount of tumor cells. We can confirm that a 5.9-mm diameter bronchoscope with a large GS can be used for lesions involving a third or less of the bronchial branch toward the target constructed by VBN.

Our study has some limitations. First, this study is not a randomized study that directly compared TBB-GS using large GS vs. small GS. However, we had performed GS-TBB using small GS for all patients up until this study. The patients characteristics between prospective group and retrospective cohort may not be well balanced although there is statistically no difference. We think that it is important to enroll continuous patients who were performed by same bronchoscopists between two period because performing by the different members will influence the result. And also, the pathological subtypes between two groups were not different. Therefore, we believe it is reasonable to compare the previous consecutive samples obtained using small GS with prospectively obtained samples using large GS. Second, in this study, two cytopathologists counted the number of cells, but artificial intelligence or other methods might be more accurate to count the number of tumor cells. Third, this study was conducted in a single institution and had a limited sample size. Fourth, we did not investigate the success rate of molecular tests because we did not performed the gene panel testing in clinical practice in most of the study period. Therefore, further multi-center studies with larger sample sizes and genetic molecular tests are needed to validate our findings of the utility of large GS.

### Conclusions

The sample size obtained with large GS was significantly larger compared to that obtained with small GS, but there was no significant difference in tumor cell number. We can use the 5.9-mm diameter bronchoscope with large GS for lesions involving a third or less bronchial generation.

## Supporting information

S1 Table Dataset for patients. (XLSX)

S1 File TREND checklist. (PDF)

S2 File Protocol (original) in Japanese. (DOCX)

S3 File Protocol in English. (DOCX)
