# Supplementary material for: Yield of tumor samples with a large guide-sheath in endobronchial ultrasound transbronchial biopsy for non-small cell lung cancer: A prospective study
Source: PLoS One. 2021 Oct 29;16(10):e0259236. doi: 10.1371/journal.pone.0259236 (PMC8555788; doi:10.1371/journal.pone.0259236)
Supplement: S2 File — (DOCX) [file pone.0259236.s003.docx]

**試験実施計画書**

**肺末梢病変に対する****気管支内腔超音波断層法における太径ガイドシースの有用性の探索的研究**

第1.6版　　2020年3月31日

| 研究責任者 |
| --- |
| 氏名：　立原素子  所属：　神戸大学大学院　医学研究科　内科学講座　呼吸器内科学分野TEL：078-382-5660  FAX：078-382-5661  E-mail：mt0318@med.kobe-u.ac.jp |

| 研究事務局 |
| --- |
| 事務局名　桂田　直子  事務局住所　神戸市中央区楠町7-5-2  TEL：078-382-5660  FAX：078-382-5661  E-mail：nk1208@med.kobe-u.ac.jp |


目次

[**1．試験の概要** 3](#_Toc514002070)

[**2．背景・目的** 4](#_Toc514002071)

[**3．試験薬又は試験機器の概要** 6](#_Toc514002072)

[**4．研究対象者** 6](#_Toc514002073)

[**5．試験の方法** 7](#_Toc514002074)

[**6．評価項目** 11](#_Toc514002075)

[**7．観察および検査項目** 12](#_Toc514002076)

[**8．中止基準** 14](#_Toc514002077)

[**9．有害事象発生時の取扱い** 14](#_Toc514002078)

[**10．試験の中止、中断または終了** 16](#_Toc514002079)

[**11．試験実施期間** 17](#_Toc514002080)

[**12．解析対象、および統計解析方法(主要解析・副次的解析）** 17](#_Toc514002081)

[**13．試験実施計画書、症例報告書又は解析計画に関する変更** 19](#_Toc514002082)

[**14．データマネジメント** 19](#_Toc514002083)

[**15．臨床試験実施のための品質保証** 19](#_Toc514002084)

[**16．倫理指針およびヘルシンキ宣言等への対応** 20](#_Toc514002085)

[**17．研究対象者の人権および安全性・不利益に対する配慮** 20](#_Toc514002086)

[**18．研究対象者に説明して同意（インフォームドコンセント）を得る方法** 22](#_Toc514002087)

[**19．研究対象者の費用負担** 23](#_Toc514002088)

[**20．記録文書等の保存** 23](#_Toc514002089)

[**21．研究計画の登録** 23](#_Toc514002090)

[**22．研究資金源および各研究者のCOI状態の開示** 23](#_Toc514002091)

[**23．研究成果の帰属と研究結果の公表** 24](#_Toc514002092)

[**24．研究組織** 24](#_Toc514002093)

**[25．文献リスト・参考資料](#_Toc514002094)** [26](#_Toc514002094)

# **1．試験の概要**

**1.1　タイトル**

肺末梢病変に対する気管支内腔超音波断層法における太径ガイドシースの有用性の探索的研究

**1.2　目的**

肺癌が疑われる肺末梢病変に対する気管支鏡検査において、太径ガイドシース(guide-sheath: GS)と細径GSとで、得られる検体中の腫瘍細胞数の差があるか、前向き登録例（全例太径GSを使用する）と後ろ向き登録例（過去に細径GSを使用した症例）との比較をすることで評価する。

主要評価項目（Primary endpoint）

　・生検鉗子で採取した検体中の腫瘍細胞数

副次評価項目（Secondary endpoint）

1) 前向き登録群と後ろ向き登録群について下記項目を評価する。

・超音波での病変の描出が可能であったか

　　　　・組織診断可能であったか

　　　　・全検体中の腫瘍組織が得られた検体数

・検体の大きさ

・PD-L1検査実施可能であったか

・検体中の有核細胞における腫瘍細胞の割合

　　 　　　　　・合併症

2) 前向き登録群のみ下記項目の評価をする。

細径GSへの変更、変更した病変の大きさ、位置、性状、関与気管支

**1.3　対象**

以下の適格基準を全て満たし、かつ以下の除外基準のいずれにも該当しない患者を、本試験の研究対象者とする。

**1.3.1 選択基準：**

1. 同意取得時の年齢が20歳以上。

2. 非小細胞肺癌が疑われる肺末梢病変に対しガイドシース併用気管支内腔超音波断層法で気管支鏡検査が行われる患者

3. 対象者本人から本研究に対して、本人の自由意志による文書同意が得られている。

**1.3.2.除外基準：**

1. 気管支鏡で可視範囲に病変を認めると思われる症例。

2. すでに治療が導入されている、再生検の症例。

3. CTなどの画像検査で明らかに良性病変と考えられる症例。

4. 重篤な合併症を有し、検査に支障をきたす可能性のある症例。

5. 抗凝固剤、抗血小板剤内服中で、一時中止できない症例。(ヘパリンへの一時変更可能な場合を除く)

6. 妊娠中の女性。

7. その他、本試験の担当者が不適当と判断した症例。

**1.4 症例数**

登録予定数:　前向き登録 80例

　　　　　　　　　太径GSで採取した検体で、非小細胞肺癌36例が得られた時点で新規登録終了とする。

後ろ向き登録：36例を想定；（前向き登録例のうち太径GSで得られた例数と同数）

登録期間:　前向き登録：倫理審査委員会承認日　～　2020年12月31日

　　　　　　　後ろ向き登録：2017年1月1日～2017年12月31日

解析期間： 2021年1月1日～2021年6月30日

**1.5　問い合わせ先**

氏名：桂田　直子

所属：　神戸大学医学部附属病院　呼吸器内科

研究事務局住所　兵庫県神戸市中央区楠町7丁目5-2

TEL：078-382-5660、FAX：078-382-5661

E-mail：nk1208@med.kobe-u.ac.jp

# **2．背景・目的**

**2.1　背景**

我が国では肺癌の罹患数、死亡数とも増加し、がん死亡原因の1位となり^1)^、肺癌診療はますます重要となっている。一方、EGFRなどの遺伝子変異を標的とした薬剤や免疫チェックポイント阻害薬といった新規治療薬が次々と登場しており、そのような治療薬が適応となる患者では飛躍的に生存期間が延長している。肺癌診療ガイドラインでは、腫瘍組織の遺伝子変異、PD-L1結果に沿った治療薬の選択が推奨されており、腫瘍組織採取の重要性が増している。免疫チェックポイント阻害薬であるペムブロリズマブは、投与前にPD-L1 陽性を組織で確認する必要があり、その評価のためには組織中に100 個以上の Viableな腫瘍細胞が必要とされ^2)^、十分な腫瘍量が必要である。

これまで、気管支鏡検査は組織診断率を上げるために様々な工夫がされてきた。ガイドシースを装着した超音波プローブを、気管支鏡の鉗子チャンネルを介して病変部付近まで挿入して、病変の位置をエコーで確認し、同部位に留置したガイドシース内に生検鉗子や細胞診ブラシ等の処置具を挿入して生検、擦過などを行うガイドシース併用気管支内腔超音波断層法（endobronchial ultrasonography with a guide sheath: EBUS-GS法）が開発され良好な診断率と安全性が示されている^3,4)^。気管支鏡、処置具の誘導に、事前に撮影したCTを用いて病変までのルートの仮想画像を使用するバーチャル気管支鏡ナビゲーション（virtual bronchoscopic navigation: VBN）を併用することによって、診断率が向上した^5)^。VBN併用EBUS-GS法での気管支鏡検査は、肺末梢病変に対する診断率が60-80%程度^6)^と有用性が確立された手技であり、肺癌診療を行う多くの施設で導入されている。

現在組織を得るためにEBUS-GS法で使用できる生検鉗子は、オリンパスメディカル社からディスポーザブルガイドシースキットとして、K-202（鉗子外径 1.5mm、適用チャンネル径 2.0mm、”細径GS”とする） とK-203　(鉗子外径1.9mm, 適用チャンネル径 2.6mm、”太径GS”とする) の2種類があり、太径GSキットで使用する鉗子は細径GSを使用するより大きな検体が採取できる。細径GSはチャンネル径2.0mmの細径気管支鏡（オリンパスメディカル社　P290 (外径4.2mm）, Q290 (外径4.8mm）など）、太径GSはチャンネル径2.8-3.0mmの気管支鏡(オリンパスメディカル社 1T260 (外径5.9mm), 1TQ290 (外径5.9mm)など) を使用する。太径GSは、得られる検体が大きいという優れた利点があるが、細径気管支鏡に比較して、特に上葉末梢の関与気管支を選択しにくいという欠点がある。　現在国内では、慣習的に細径GSが使用されていることが多く、また、太径GSを積極的に使用している施設においても、どちらのGSを選択するかは、明確な基準はなく術者の判断に委ねられている。　肺結節影(腫瘍径3cm以下)に対して全例に太径GSを使用した検討では、診断率　74.4 %(悪性腫瘍に限ると83.3%)と報告され^7)^、これまでの細径GSを用いた検討と同等の診断率であった。肺結節影に対するVBN併用EBUS-GS法の診断率の検討では、GSの選択は術者の判断で決定されていたが、そのサブ解析においてGSの太さの違いによる診断率には差はなかったと報告された^8)^。　このように報告数は少なく、直接比較はされていないものの、太径GSの診断率は細径GSに遜色はないものと考えられるが、得られた検体中の腫瘍細胞数の検討はこれまでなされていない。PD-L1検査など治療選択に必要な検査をすすめるためには、できるだけ多くの腫瘍細胞を採取することが必要である。実際、当科ではこれまで細径GSを使用してきたが、検査に提出したうちの約3%の症例で検体採取量が不十分であり、腫瘍細胞数が少ないために再度気管支鏡検査を行うこともあった。

**2.2　試験の目的**

**2.2.1　主要目的**

　肺癌が疑われる肺末梢病変に対する気管支鏡検査において、太径GSを用いた方が、より多数の腫瘍細胞の採取が可能であることを評価する。

**2.2.2　副次目的**

・細径GSへの変更率、変更病変の大きさ、位置、性状、関与気管支を評価する。

臨床的意義：太径GS使用で診断が難しい病変を明らかにすることで、GSの太さの選択が容易になる。

・太径GSと細径GSの病変描出率を評価する。

　　（超音波で病変が描出可能であった症例を分子、太径GSと細径GSそれぞれの全例を分母とする。）

・太径GSと細径GSの組織診断率を評価する。

　　（組織診断が可能であった症例を分子、太径GSと細径GSそれぞれの全例を分母とする。）

・太径GSと細径GSの得られた全検体中の腫瘍組織が得られた検体数を評価する。

・太径GSと細径GSの検体の大きさを評価する

・太径GSと細径GSのPD-L1発現検索可能割合を評価する。

・太径GSと細径GSの検体中の有核細胞における腫瘍細胞の割合を評価する。

・太径GSと細径GS施行例の出血や気胸など合併症の差を評価する。

**2.2.3　探索的目的**

該当せず。

# **3．試験薬又は試験機器の概要**

**3.1 試験薬（試験機器）情報**

オリンパスメディカル社　ディスポーザブルガイドシースキット

|  | K-202(細径GS) | K-203(太径GS) |
| --- | --- | --- |
| ガイドシース | SG-200C | SG-201C |
| 外径 | 1.95mm | 2.55mm |
| 生検鉗子 | FB-233D | FB-231D |
| 外径 | 1.5mm | 1.9mm |
| 細胞診ブラシ |  | BC-202D-2010 |
| 外径 |  | 1.8mm |

　使用方法：気管支鏡の鉗子チャンネルを介してガイドシースを病変部付近まで挿入して、病変の位置をエコーで確認し、同部位に留置したガイドシース内に生検鉗子や細胞診ブラシ等の処置具を挿入して生検、擦過などを行う。K-202キットには、細胞診ブラシが添付されていないが、BC-202D-2010を使用する。

いずれのキットとも保険適応内である。

**3.2 予期される有害反応（医療機器の場合は予期される不具合）**

市販後広く使用されている機器であり、安全性は担保されているが、稀に機器の破損、生検鉗子が開かないなど機能の低下、ガイドシースの X 線不透過チップの位置ずれ、脱落の可能性がある。

# **4．研究対象者**

以下の適格基準を全て満たし、かつ以下の除外基準のいずれにも該当しない患者を、本試験の研究対象患者とする。

**4.1 選択基準：**

以下の基準を全て満たす患者を対象とする。

(1) 同意取得時の年齢が20歳以上である。

(2)　非小細胞肺癌が疑われるが確定診断に至っていない肺末梢病変に対してEBUS-GS法で気管支鏡検査が行われる患者

(3) 対象者本人から本研究に対して、本人の自由意志による文書同意が得られている。

設定根拠：

(1) 法律上個人の同意が成立する年齢を考慮して設定した。

(2) 本研究の対象を特定するために設定した。

(3) 倫理的な配慮のため設定した。

**4.2 除外基準：**

以下のうち１つでも該当する患者は対象として除外する。

1. 気管支鏡で可視範囲に病変を認めると思われる症例。
2. すでに治療が導入されている、再生検の症例。
3. CTなどの画像検査で明らかに良性病変と考えられる症例。
4. 重篤な合併症を有し、検査に支障をきたす可能性のある症例。

コントロール不良な狭心症、高血圧症、気管支喘息、3ヶ月以内の心筋梗塞などの重篤な心疾患、重症感染症、凝固異常など。

(5) 抗凝固剤、抗血小板剤内服中で、一時中止できない症例。(ヘパリンへの一時変更可能な場合を除く)

(6) 妊娠中の女性。

(7) その他、本試験の担当者が不適当と判断した症例。

設定根拠：

(1)(2)(3)有効性評価に影響を及ぼす可能性があるため設定した。

(4)(5)(6) 検査の安全性を保つため、本研究の対象として不適当な患者を除くために設定した。

(7) 研究対象者に対する安全性を確保する上で、また適正に試験を実施する上で、本研究の対象として不適当な患者を除くために、担当者が検討できる余地を残すために設定した。

# **5．試験の方法**

**5.1 試験の種類・デザイン**

　　前向き介入試験（ヒストリカル・コホート解析）

　　　　上記で得られた結果と、細径ガイドシースのヒストリカル・コホート解析を行う。

　　設定根拠：当科では、2017年12月までは3例の太径GS使用例を除き、全例細径GSを使用してきた。前向き対象群では、全例太径GSを使用するため、過去の連続した細径GS使用例をヒストリカル・コントロールとすることは妥当である。

**5.2 試験のアウトライン（試験のフローチャート参照）**

本試験は、肺末梢病変を有する患者を対象に、太径GSの有用性を検証するための単施設、ヒストリカル・コントロール比較試験である。

肺末梢病変に対してガイドシース併用気管支内腔超音波断層法を行う、80名の研究対象者(前向き登録群)に対して、全例太径GS 使用する。太径GSで超音波での病変の描出が困難な場合、細径GSに変更して検体採取を行う。また2017年1月1日～2017年12月31日までに肺末梢病変に対してガイドシース併用気管支内腔超音波断層法を行った細径GS使用の連続症例を、前向き登録例のうち太径GSで検体採取できた症例と同数(36例に想定)を登録(後ろ向き登録群)する。前向き登録群と後ろ向き登録群との得られた腫瘍細胞数の差を主要評価項目として検討する。試験期間は、気管支鏡検査前検査日、気管支鏡検査当日、気管支鏡検査結果説明日の合計3日間である。試験スケジュールの概略を図1に示す。

病理

組織診断

BF

本登録

病理検体評価

（腫瘍細胞数）

適格性

確認

・前向き登録群

・後ろ向き登録群

登録

1次登録

BF前検査

同意取得

・後ろ向き登録群

病理検体評価

（腫瘍細胞数）

**5.3 症例登録、割付方法**

研究責任者または研究分担者は、研究対象者からの同意取得後、試験治療開始に至るまでを以下の手順に従う。

1. 研究責任者または研究分担者は、研究対象者からの同意取得後、スクリーニング検査を行い、研究対象者の適格性を判定する。なお、研究対象者の同意が得られた場合、同意取得前の検査結果を用いてもよい。
2. 研究責任者または研究分担者は、研究対象者が適格基準をすべて満たし、除外基準のいずれにも該当しないことを確認した上で、「症例登録票」に必要事項をすべて記入し、研究事務局内の定められたBoxに入れるか、手渡しをする。Faxでの送付はしない。

研究対象者の特定を容易にするため、研究対象者識別番号リストを作成する。

＜症例登録票提出先＞

研究事務局

〒650-0017 神戸市中央区楠町7-5-1

TEL：078-382-5660

FAX：078-382-5661

E-mail： nk1208@med.kobe-u.ac.jp

受付時間：　10：00 - 17：00　（土日祝日はのぞく）

1. 研究事務局では適格性を確認し、判定結果及び登録番号を記した「症例登録確認書」を発行する。(一次登録)
2. 研究責任者または研究分担者は、「症例登録確認書」の判定結果を確認し、試験治療を開始する。「症例登録確認書」を受領するまで試験治療を開始してはならない。気管支鏡検査による内腔観察により、二次登録（本登録）を行う。
3. 「症例登録票」及び「症例登録確認書」は、原資料として適切に保管する。

| 同意取得 | ⇨ | スクリーニング検査 | ⇨ | 適格性の確認 | ⇨ | 症例登録票の記入 | ⇨ | 一次症例登録 | ⇨ | 症例登録確認書の受領 | ⇨ | 検査・本登録 | ⇨ | 試験開始 |
| --- | --- | --- | --- | --- | --- | --- | --- | --- | --- | --- | --- | --- | --- | --- |

**5.4試験治療**

肺末梢病変に対してガイドシース併用気管支内腔超音波断層法を行う。全例太径GS 使用するが、太径GSで超音波での病変の描出が困難な場合には、細径GSに変更して検体採取を行う。

1. 検査前

仮想画像作成

CTの再構成データ（DICOM形式）をBf-NAVI (オリンパスメディカルシステム社)に読み込み、目標までの仮想画像を作成する。関与気管支に至るルートが容易に判断できる場合には、Bf-NAVIは必ずしも必要としない。

標的とする腫瘍のサイズ、位置、関与気管支の有無、性状を確認する。

画像および腫瘍マーカー結果の確認をし、小細胞肺がんの可能性が非常に高い場合は、二次

登録(本登録)しない。登録しない場合は、日常診療としての気管支鏡検査を継続する。

2. 検査

1) 前処置

4%キシロカインビスカスによる咽喉頭麻酔後、4%キシロカインをJackson’s sprayで噴霧する。

適宜ヒドロキシジン塩酸塩 25 mg筋注、もしくは、ミダゾラムの間欠的静注を行う。

2) 気管支鏡挿入、表面麻酔、内腔観察

気管支鏡（オリンパスメディカルシステムズ社、1TQ290, 1T260）を挿入し、気管分岐部を初めとして各分岐部で、2％キシロカインによる表面麻酔を行う。2％キシロカイン2 mlを各分岐部で2回程度噴霧しすぐに余分なキシロカインと分泌物を吸引する。各区域支に表面麻酔を行ったのち、気管支内腔観察を行う。腫瘍の明らかな露頭や責任気管支の狭窄がないことを確認し、二次登録（本登録）とする。登録しない場合には、試験に登録せずに日常診療としての気管支鏡検査を継続する。

　　　3) 病変の確認およびGS留置

VBN像の情報もしくは**CT**画像情報に従って関与気管支に気管支鏡を挿入し、太径GS(SG-201C)を装着した超音波プローブを鉗子孔に挿入し、X線透視下に病変の位置を確認しながら、病変をEBUSで確認する。太径GS超音波プローブは外径2.0mmで20MHzメカニカルラジアル型走査方式のもの(XUM-S20-20R)を用いる（すべてオリンパスメディカルシステムズ社製）。太径GS挿入後、病変の描出ができない場合は、キュレット型誘導子を使用するか、細径GS(SG-200C)に変更して病変の描出を行う。細径GSへ変更する際には、気管支鏡を細径気管支鏡（P290, Q290, P260F）へ変更し、細径GS超音波プローブ(XUM-S20-17S,外径1.7mm)を用いて病変をEBUSで確認する。病変部の描出後、GSを病変内に留置して、超音波プローブは抜去する。

4) 生検

ガイドシース内に生検鉗子を挿入し、病変部にて鉗子による組織診およびブラシ擦過細胞診を行う。生検により組織を5個採取する。ブラシ擦過細胞診は2回施行し、生検およびブラシを交互に行う。その後、必要に応じてTBNA針やキュレット、通常鉗子などでの検体採取の追加を行う。

GSを引き抜く際は、GSを病変部位に留置したまま、20mlのシリンジで20秒の持続吸引を行い、その後GSを引き抜く。

3.検査後 (検体処理)

組織生検検体はヘマトキシリン・エオジン染色と必要があれば特殊免疫染色を行い、病理学的診断を行う。腫瘍細胞数の計測は、1名の病理医、1名の細胞診専門医が行う。擦過細胞診検体はパパニコロウ染色を行い細胞診学的診断を行う。生検鉗子および擦過ブラシ付着細胞は生理食塩水で洗浄し、細胞診・一般細菌および抗酸菌の塗沫、培養検査を行う。また、GSを介して吸引した検体は、必要に応じてデバイス洗浄生理食塩水と混合しセルブロックを作成する。

**5.5 併用薬(療法)に関する規定**

該当せず

**5.6 休薬の方法**

該当せず

**5.7 試験機器の管理・交付手順**

保険診療の肺末梢病変に対する気管支鏡検査として用いるため、試験に際して特別な管理はない。

**5.8 服薬指導情報**

該当せず

**5.9 後治療**

該当せず

**5.10 試験終了後の対応**

太径GSで検体採取が困難である場合、細径GSに変更して検体採取を行うため、これまで行っている気管支鏡検査と比較して診断率が低い可能性は低い。しかし、確定診断に至らなかった症例については、気管支鏡検査の再検もしくは確定診断に至る他の検査（CTガイド下肺生検、胸腔鏡下肺生検）を勧める。やむを得ず経過観察をする場合は最大2年間観察し、増大がなければ非腫瘍性と判断する。

# **6．評価項目**

**6.1 主要評価項目（Primary endpoint）**

生検鉗子で採取した検体中の腫瘍細胞数

**6.2 副次評価項目（Secondary endpoint）**

**6.2.1　有効性評価項目**

　・前向き登録群と後ろ向き登録群について下記項目を評価する。

(1) 超音波で病変の描出が可能であったか

　　(超音波で病変の描出が可能であったかを評価する)

(2) 組織診断可能であったか

　　（組織診断を得ることができたかを評価する。）

(3) 全検体中の腫瘍組織が得られた検体数

　　（全検体のうちに腫瘍組織を得ることのできた検体数について評価する。）

(4) 検体の大きさ

(5) PD-L1検査実施可能であったか

(6) 検体中の有核細胞における腫瘍細胞の割合

**6.2.2　安全性評価項目**

　　 合併症

　　（出血、気胸、感染、その他の合併症について両群で評価する。）

**6.2.3 その他の評価項目**

・前向き登録群のみ下記項目の評価をする。

細径GSへの変更、変更した病変の大きさ、位置、性状、関与気管支

# **7****．観察および検査項目**

**7.1 実施スケジュールと手順**

観察・検査・評価の実施スケジュールを以下の表に示す。研究責任者又は研究分担者は、スケジュールに従って観察・検査等を実施する。

| 日程 |  | 気管支鏡検査前  （スクリーニング検査）  Visit 1 | 検査中  Visit 2 | 検査終了後  (病理検査判明時)  Visit 3 |
| --- | --- | --- | --- | --- |
| 検査 項目 | 胸部単純X線写真  血液検査（血算・生化学・凝固・感染）  心電図  呼吸機能検査  胸部CT  気管支鏡検査  病理検査 | ○  〇  〇  〇  〇 | 〇 | 〇 |
| 観察 項目 | 研究対象者の背景  バイタルサイン  腫瘍サイズ・位置  挿入可能分枝数  細径GSへの変更  病変描出の程度  生検回数  検体個数  合併症  検体中腫瘍細胞数  組織診断  検体の大きさ  腫瘍組織検体数  PD-L1検査  検体中腫瘍細胞割合 | 〇  〇  〇 | 〇  ○  〇  〇  〇  〇  ○ | 〇  〇  〇  〇  〇  〇  〇 |

なお、本試験に関わるデータは、同意取得前のデータも使用する場合があるが、予め研究対象者に同意を取得した上で採用する。

**7.1.1 スクリーニング検査**

研究責任者又は研究分担者は以下のスクリーニング検査を行い、選択基準を満たし、除外基準に抵触しない患者を研究対象者とする。検査項目は以下に記載の通りとする。

1. 研究対象者の背景の調査　（現病歴、既往歴、併存症、処方薬、アレルギーの有無）
2. バイタルサイン(心拍数・血圧・体温・SpO_2_)
3. 血液学的検査(WBC、RBC、Hb、Hct、Plt、白血球分画), 凝固系(PT, APTT)
4. 生化学検査(AST、ALT、T-BIL、TP、ALB、BUN、CRP、Na、K、Cl、Cr)
5. 心電図
6. 胸部単純X線写真
7. 呼吸機能検査

**7.1.2 研究対象者の情報**

同意取得時又はスクリーニング検査時に、以下の研究対象者情報を記録する。

　1) 同意取得日

　2) 研究対象者識別コード

　3) 研究対象者背景

・性別 ・同意取得時年齢

・身長 ・体重

・既往歴 ・併存症　・処方薬　・喫煙歴

**7.1.3 観察・検査・評価項目**

Visitごとの検査項目を以下に記す。

**Visit 1 気管支鏡検査前**

(日常診療で気管支鏡検査前の検査として施行している項目であるため、スクリーニングと同日に行う。)

1. 胸部CT

・腫瘍サイズ

・位置

・関与気管支の有無

・性状

**Visit 2 気管支鏡検査当日**

　　　　　　①　気管支鏡検査

　　　　　　　　 ・気管支鏡挿入可能分岐数

　　　　　　　　 ・太径EBUS 所見

　　　　　　 ・細径GSへの変更の有無

　　　　　　　　　・細径GS へ変更した場合は、細径EBUS所見

・生検回数

　　　　　　 ・検体個数

　　　　　　　　　・予防的抗菌薬の有無

　　　　　　 ・合併症の有無

**Visit 3 気管支鏡検査結果説明時**

①　合併症の有無

**(Visitなし)　病理検査検討**

①　腫瘍細胞数

②　検体の大きさ

③　組織診断

④ PD-L1検査可能であったか

⑤　検体中の有核細胞における腫瘍細胞の割合

**7.2他機関への試料・情報の提供**

提供は行わない。

**7.3 実施計画書からの逸脱の取扱い**

研究責任者または研究分担者は、研究倫理審査委員会の事前の審査に基づく研究機関の長の承認を得る前に、試験実施計画書からの逸脱あるいは変更を行わない。

研究責任者または研究分担者は、緊急回避等のやむを得ない理由により、研究倫理審査委員会の事前の承認を得る前に、試験実施計画書からの逸脱あるいは変更を行うことができる。その際には、研究責任者または研究分担者は、逸脱または変更の内容および理由ならびに試験実施計画書等の改訂が必要であればその案を速やかに研究倫理審査委員会に提出し、研究倫理審査委員会および研究機関の長の承認を得る。

研究責任者または研究分担者は、試験実施計画書からの逸脱があった場合は、逸脱事項をその理由とともに全て記録する。

研究責任者または研究分担者は、本試験について人を対象とする医学系研究に関する倫理指針に適合していないこと（適合していない程度が重大である場合に限る。）を知った場合には、速やかに研究機関の長に報告し、必要な対応をした上で、その対応の状況･結果についての研究機関の長による厚生労働大臣等への報告･公表に協力する。

# **8****．中止基準**

研究責任者または研究分担者は、以下に示す理由で試験継続が不可能と判断した場合には、試験を中止し、中止・脱落の日付・時期、中止・脱落の理由、経過をカルテならびにCRFに明記するとともに、中止・脱落時点で必要な検査を行い有効性・安全性の評価を行う。

①　　研究対象者から試験参加の辞退の申し出や同意の撤回があった場合

②　　登録後に適格性を満足しないことが判明した場合

③　　有害事象により検査の継続が困難な場合

④　　試験全体が中止された場合

⑤　　その他の理由により、研究責任者または研究分担者が試験を中止することが適当と判断した場合

# **9．有害事象発生時の取扱い**

**9.1 有害事象の定義**

有害事象とは気管支鏡検査に伴い生じる、好ましくない、あるいは意図しない徴候（臨床検査値の異常変動を含む）、症状または疾病のことであり、検査との因果関係を問わない。

**9.2 有害事象発生時の研究対象者への対応**

研究責任者または研究分担者は、有害事象を認めた時は、直ちに適切な処置を行うとともに、カルテならびに症例報告書に齟齬なく記載する。また、検査を中止した場合や、有害事象に対する治療が必要となった場合には、研究対象者にその旨を伝える。

**9.3 報告の対象となる有害事象**

検査が開始されてから終了までに発生したすべての有害事象は検査との因果関係の有無に関わらず報告し、有害事象が消失するか固定するまで観察する。また、検査との因果関係があると判断された有害事象については試験期間終了時まですべて報告する。

**9.4 有害事象発生時の報告手順**

上記期間に発生したすべての有害事象について、研究責任者または研究分担者は、カルテならびに症例報告書に齟齬なく記載する。

**9.5 有害事象の評価に必要な記載内容**

有害事象の重症度評価は、①軽度：検査中止のみで無処置で軽快する場合、②中等度：検査の中止および何らかの処置が必要であるが、重症ではない場合、③重度：検査の中止および入院が必要な状態もしくは生命の危険がある状態と定義する。

・有害事象の名称

・発現日

・転帰日

・転帰：回復、軽快、後遺症あり、未回復、死亡、不明

・処置（試験薬の投与）：変更なし、中止、休薬、減量、増量、該当せず

・重症度：軽度、中等度、重度

・試験薬との因果関係：関連あり、関連なし

**9.5.1 有害事象の回復性と試験薬との因果関係**

有害事象の回復とは、有害事象がない状態、又は投与前の状態への改善とする。有害事象における検査との因果関係の判定に際しては、研究対象者の全身状態、合併症、併用薬・併用療法、時間的関係を勘案して判断する。

**9.6 重篤な有害事象発生時の取り扱い**

**9.6.1 重篤な有害事象の定義**

重篤な有害事象とは、次のいずれかに該当するものとする。

（1） 死亡

（2） 死亡につながるおそれのあるもの

（3） 障害（日常生活に支障をきたす程度の機能不全の発現）

（4） 障害につながるおそれのあるもの

（5） 治療のために病院又は診療所への入院又は入院期間の延長が必要とされるもの

（6） （1）～（5）までに掲げる症例に準じて重篤であるもの

**9.6.2 報告の対象となる重篤な有害事象**

試験期間中の全ての重篤な有害事象、および試験終了（中止）後に気管支鏡検査との関連性が疑われる重篤な有害事象について報告する。

**9.6.3 重篤な有害事象の報告手順**

有害事象が発生し、研究責任者等が重篤と判断した場合、次の手順に従い当該有害事象情報を取り扱う。

1. 研究責任者から研究機関の長及び研究責任者への報告

研究責任者は、因果関係に関わらず、当該有害事象情報を可能な限り速やかに実施研究機関の長及び研究責任者に報告する。報告は第1報（緊急報告）および第2報（詳細報告）とする。

1. 厚生労働大臣への報告

人を対象とする医学系研究に関する倫理指針に基づき、研究機関の長が、厚生労働大臣への報告が必要と判断した場合には、研究機関の長は、「予期しない重篤な有害事象報告」（厚生労働省指定の別添の様式に準ずる）を作成し、厚生労働大臣に報告する。

1. 追加情報の入手時の対応

当該有害事象が発生した研究機関の研究責任者は、当該事象に関する追加情報が得られた場合には、可能な限り速やかに研究機関の長に追加報告を行う。当該追加情報の取扱いは、(1), (2)の手順に準ずる。

1. 医薬品・医療機器等安全性情報報告制度に基づく対応

市販後の薬剤や医療機器については医薬品・医療機器等安全性情報報告制度に基づいて対応し、必要に応じて厚生労働省に報告する。

# **10．試験の中止、中断または終了**

**10.1 試験全体での中止または中断の基準**

研究事務局は、以下の情報が得られ、試験全体の続行が困難であると考えられる時には、研究責任者と試験全体の中止又は中断について協議のうえ、決定する。

- 1. 予期できない重篤な有害事象の発生
  2. 予期できる重篤な有害事象の発生件数、発生頻度、発生条件等の発生傾向が添付文書から予測できないことを示す情報
  3. 重篤な有害事象のうち因果関係がないと判断されていたが、その後発生数、発生頻度、発生条件等の発生傾向から因果関係が否定できないと判断される情報
  4. 重大な障害もしくは死亡が発生するおそれがあることを示す研究報告
  5. 当該研究で有効性が認められないことを示唆する情報

**10.2 試験全体での中止又は中断する場合の手続き**

研究責任者は試験全体を中止又は中断する場合には、研究機関の長にその旨とその理由を詳細に速やかに文書で通知する。また、投与中の研究対象者に対して速やかにその旨を伝え、適切な治療への変更等の適切な処理を行うものとする。

**10.3 個々の実施医療機関での試験を中止または中断する場合の手続き**

研究責任者は、試験を中止又は中断した場合には、研究機関の長に速やかにその旨を文書で通知するとともに、中止又は中断について文書で詳細に説明する。

**10.4 試験の終了**

研究責任者は、試験終了後、研究機関の長に試験が終了した旨を文書で通知し、試験結果の概要を文書で報告する。

# **11．試験実施期間**

前向き症例登録

倫理審査委員会承認日　～　2020年12月31日

後ろ向き症例登録

2017年1月1日～2017年12月31日

解析期間： 2021年1月1日～2021年6月30日

# **12．解析対象、および統計解析方法(主要解析・副次的解析）**

本試験の統計解析計画の概要を以下にまとめた。

統計解析計画書において本試験実施計画書の概要を修正することがあるが、主要評価項目の定義や解析方法が変更される場合には、本試験実施計画書を改訂する。

**12.1 解析対象集団**

**12.1.1 最大の解析対象集団 (full analysis set：FAS)**

本試験に登録され、気管支鏡検査を施行され、有効性データがあるすべての研究対象者を最大の解析対象集団 (FAS) とする。ただし、重大な試験実施計画書違反 (同意未取得、期間外の登録等) の研究対象者については除外する。

**12.1.2 試験実施計画書に適合した対象集団 (per protocol set：PPS)**

FAS から、試験方法など試験実施計画書の規定に対して、以下の重大な違反があった症例を除いた研究対象者とする。

・選択基準違反

・除外基準違反

**12.1.3 安全性解析対象集団**

該当せず。

**12.2 目標症例数と設定根拠**

目標症例数：太径GS(前向き登録)：80例

太径GSで採取した検体で、非小細胞肺癌36例が得られた時点で新規登録は終了とする。

細径GS(後向き登録) 36例を想定（実際に太径GSで生検できた症例と同数）

【設定根拠】

　本研究の主要評価項目である検体中の腫瘍細胞数に関して、これまで太径GSと細径GS を使用して得られる検体中の腫瘍細胞数を検討した報告はない。当科で施行した2症例の太径GS の1プレパラートあたりの平均腫瘍細胞数は、1410個, 302個で、3症例の細径GSでは、192個, 675個, 407個であった。2群間の平均の差が431, 標準偏差 855であり、αエラー20％、検出力80％のもとで解析すると必要症例数は36例と見積もられる。細径GSへの変更例、若干の解析除外例の発生を考慮し、本試験の前向き登録の目標症例数は80例とした。太径GSで採取した検体で、非小細胞肺癌36例が得られた時点で試験への新規登録を終了する。細径GS使用の後ろ向き登録は、前向き登録例数と同数とする。EZR®を用いて計算した。

**12.3 症例の取り扱い**

原則として登録された症例については、研究事務局および研究責任者が協議の上、症例の取り扱いを決定する。新たな問題が起こった場合の症例の取り扱いについても、および研究事務局が、協議の上、決定する。

**12.4 データの取り扱い**

データ集計・解析時におけるデータの取り扱いについて、疑義が生じた場合は、統計解析責任者と研究責任者が協議の上決定する。

**12.5 統計解析項目および解析計画**

全ての症例において気管支鏡検査が終了し、データが固定された後に解析を行う。全ての有効性評価において、最大の解析対象集団 (FAS) における解析を主解析とし、参考として試験実施計画書に合致した解析対象集団 (PPS) における解析を行う。

**12.5.1 研究対象者背景の集計**

各解析対象集団における研究対象者背景データの分布及び要約統計量を群ごとに算出する。

名義変数については、カテゴリの頻度及び割合を群ごとに示す。

連続変数については要約統計量 (例数、平均値、標準偏差、最小値、中央値、最大値) を群ごとに算出する。

群間の比較には、名義変数については、Pearson のカイ 2 乗検定、ただし期待度数が 5 未満のセルが 20% 以上の場合は Fisher の直接確率計算法、連続変数については t 検定を用いる。有意水準は両側 5%とする。

**12.5.2 有効性の解析**

**12.5.2.1 主たる解析**

太径GSと細径GSで得られた検体中の腫瘍細胞数についてt検定を用いて解析する。

有意水準は両側 5%とする。

**12.5.2.2 副次解析**

主たる解析結果を補足する考察を行う目的で有効性の副次評価項目の解析を行う。

仮説検定の有意水準は両側5%とする。

**12.5.3 安全性の解析**

太径GSと細径GS施行例の出血や気胸など合併症の発現率を比較する。

**12.5.4 中間解析**

本試験において中間解析は行わない。

**12.6 独立データモニタリング委員会**

独立データモニタリング委員会は設置しない。

**12.7 最終解析**

すべての気管支鏡検査および病理検査終了後、データが得られ症例が固定された後に解析を行う。

# **13．試験実施計画書、症例報告書又は解析計画に関する変更**

**13.1. 試験実施計画書および症例報告書の改訂**

試験実施計画書及び症例報告書を改訂する場合には、以下の手順により行う。

1. 研究責任者は試験実施計画書改訂版及び症例報告書改訂版を速やかに研究機関の長に提出し、研究機関の長を経由して速やかに研究倫理審査委員会の承認を得る。
2. 研究倫理審査委員会の意見に基づく研究機関の長の指示が研究責任者および試験調整委員会の許容できる範囲内で、試験実施計画書及び症例報告書用紙を修正する場合も同様の手順とする。

**13.2. 統計解析計画の変更**

統計解析責任者は、統計解析計画書の内容を変更した場合、変更内容をすべて本試験の統計解析報告書に記載する。なお、統計解析計画書の変更は、その経緯を記録に残す。

# **14．データマネジメント**

**14.1** **症例報告書（Case Report Form：CRF）の作成**

研究責任者または研究分担者は、研究対象者ごとに症例報告書（CRF）を作成する。

一度記入したCRFを修正する場合は、元の記載が読めるように二重線で訂正した上で修正内容及び修正日を記入し、修正者の署名または押印を残す。また、誤記訂正等の軽微な修正に該当しない場合は、修正理由をあわせて記入する。

作成したCRFに誤りがないことを確認した上で研究責任者が署名を行う。

CRF原本は、研究事務局で保管する。

**14.2 症例報告書に直接記載され、かつ原資料(原データ)とする資料の特定**

本試験においては、以下の文書などを原資料(原データ)とする。

1) 研究対象者の同意及び研究対象者への情報提供に関する記録

診療録、看護記録、臨床検査データ及び画像検査フィルム、気管支鏡検査報告書等症例報告書作成の基となった記録。なお、電子カルテに格納されたデータも原資料とみなす。

2) 本試験に関連する指針上必要な試験に係る文書又は記録

# **15．臨床試験実施のための品質保証**

**15.1 品質管理**

本試験が安全に、かつ実施計画書に従って実施されているか、データが正確に集積されているかを定期的に確認する目的でモニタリングを行う。

**15.1.1 モニタリング責任者及びモニターの指名**

研究責任者は、当該研究のモニタリング責任者及びモニターを指名する。なお、モニタリング責任者及びモニターは、「人を対象とする医学系研究に関する臨床研究に関する倫理指針」等の規制要件に関する教育履歴を有し、本研究の試験実施計画書、研究対象者の同意説明文書及びモニタリング手順書の内容を十分に理解している者から指名する。

**15.1.2 モニタリングの実施**

**15.1.2.1 症例に関するモニタリング**

モニターは、研究実施中に神戸大学医学部附属病院で、原資料等（同意書・診療録・症例報告書等）の直接閲覧を行う。

**15.1.2.2 症例以外のモニタリング**

　　　　　　　　症例以外のモニタリングは実施しない。

**15.2. 品質保証**

本試験は少数例で実施する探索的な試験であることから、監査は実施しない予定である。

# **16．倫理指針およびヘルシンキ宣言等への対応**

本試験は、ヘルシンキ宣言、人を対象とする医学系研究に関する倫理指針、および神戸大学の利益相反マネジメント指針を遵守して実施する。

# **17．研究対象者の人権および安全性・不利益に対する配慮**

**17.1 人権への配慮（個人情報の保護）**

研究責任者または研究分担者は、プライバシーの侵害に対する研究対象者の権利保護の原則を遵守する。また、関係者は、研究対象者の個人情報及びプライバシー保護に最大限の努力を払い、本試験を行う上で知り得た個人情報を正当な理由なく漏らしてはならない。関係者がその職を退いた後も同様とする。本試験では、研究対象者識別番号リストにより本試験データベース及び試験関連文書と研究対象者の原データを関連付ける。研究対象者の特定及び研究対象者識別番号リストの正確性確認のため、すべての適用される法令及び規則の範囲内で、性別、生年月日等の限定的な研究対象者の情報を用いることができる。

研究責任者または研究分担者は、データ管理を行う際、特定の個人を識別しうる記述等（氏名、イニシャル、住所、電話番号、カルテ番号等）を削除し、匿名化を行う。症例登録及び症例報告書等の作成の際には、研究対象者識別番号を用いる。なお、匿名化する際の研究対象者識別番号リストは、研究責任者が「20.記録文書等の保存」に従い保管・管理する。研究対象者識別番号リストについて、外部への提供は行わない。

研究責任者等が研究で得られた情報を公表する際には、研究対象者が特定できないよう十分に配慮する。

**17.2研究対象者に生じる負担、予測されるリスク（起こりうる有害事象を含む）**

**17.2.1　予測される利益**

本研究で太径GSを使用して検体採取した結果、得られる腫瘍細胞数が多いという利益が生じることが期待される。また、研究成果により将来の気管支鏡検査方法の進歩に貢献できる可能性がある。

**17.2.2　予測される不利益（負担及びリスク）**

本研究の研究対象者は、もともと日常診療において、肺末梢病変の診断のためにガイドシース併用気管支内腔超音波断層法での気管支鏡検査を必要としており、日常診療での気管支鏡検査の合併症を大きく超えるリスクが生じる可能性は低い。前向き登録群で太径GSでの検体採取が困難で細径気管支鏡へ変更を行う場合、検査時間が若干長くなり、気管粘膜の腫脹をきたす可能性があり、できる限り迅速に細径気管支鏡への変更を行う。

一方、細径GSの情報を用いる研究対象者に特に不利益は生じない。

**17.3 安全性・不利益への配慮**

本試験で被る有害事象のリスクや不利益を最小化するために、「選択基準」、「除外基準」について慎重に検討されている。発生した有害事象が予期された範囲内であるか否かをモニタリングするとともに、重篤な有害事象や予期されない有害事象が生じた場合には慎重に検討・審査され、必要な対策が講じられる。本研究は、保険適応範囲内で行うため、特別な補償はなく合併症が発現したときは、保険診療で対応する。重篤な有害事象が発生した場合、担当医は被験者に対して誠実な医療を提供する。

本試験による健康被害に対して行われた治療に対する費用は、原則として健康保険と患者負担により支払われる。

**17.4　研究機関の長への報告**

研究責任者は以下について文書により研究機関の長に報告する。なお、①については、年1回の報告を行い、②以降の項目は、適宜報告する。

① 研究の進捗状況

② 研究の倫理的妥当性若しくは科学的合理性を損なう事実若しくは情報又は損なうおそれのある情報であって研究の継続に影響を与えると考えられるものを得た場合

③ 研究の実施の適正性若しくは研究結果の信頼を損なう事実若しくは情報又は損なうおそれのある情報を得た場合

④ 研究が終了(停止・中止)した場合

⑤ 研究に関連する情報の漏えい等、研究対象者等の人権を尊重する観点又は研究の実施上の観点から重大な懸念が生じた場合

⑥　その他

**17.5　遺伝子変異に関する情報の開示に関する考え方と偶発的所見（Incidental Findings）**

　本試験から遺伝的特徴に関する重要な知見が得られる可能性は無い。

**17.6 遺伝カウンセリングについて**

　本試験はヒトゲノム・遺伝子解析研究に関する倫理指針の対象外として実施するため、遺伝カウンセリングは実施しない。

**17.7 データ・試料の二次利用について**

本試験で得られた研究対象者のデータはデータベース化され、今後、他の研究に用いる可能性がある。他の研究を行う際は、新たに研究計画をたて、倫理審査委員会の承認を得て実施する。その際も個々の研究対象者に対し文書による同意を求めることは行わないが、研究に関する情報公開を適切に行う。

# **18．研究対象者に説明して同意（インフォームドコンセント）を得る方法**

**18.1同意文書及びその他の説明文書の作成並びに改訂**

研究責任者または研究担当者は、研究対象者から試験参加の同意を得るために用いる同意文書及びその他の説明文書を可能な限り平易な表現で作成する。また、同意説明文書及びその他の説明文書を改訂する必要があると認めた場合は、これらを改訂する。研究責任者または研究担当者は、研究対象者候補には質問する機会、および同意するかどうかを判断するための十分な時間を与え、本試験の内容を良く理解したことを確認した上で、自由意思による同意を得る。

研究責任者または研究担当者は、作成又は改訂された同意説明文書及びその他の説明文書を介入研究倫理審査委員会に提出し、その承認を得る。

**18.2　代諾者等からインフォームド・コンセントを受ける場合の手続き**

本研究における前向き登録群は研究対象者本人から同意を得るものとする。

後ろ向き登録群については、死亡例やその他連絡不能である症例も含まれるため、研究対象者個々からインフォームド・コンセントを得ることができない。そのため、情報公開文書をインターネット上に公開し、研究対象者が研究登録への同意を拒否できる機会を設ける。

**18.3　インフォームド・アセントを受ける場合の手続き**

該当せず。

**18.4　研究対象者およびその代諾者に対する説明事項**

研究責任者が作成する説明文書には、以下の事項を記載する。

1.はじめに（この試験が研究を伴うこと）

2.この臨床試験の目的

3.あなたに研究参加をお願いする理由

4.この臨床試験の方法（治療内容）や期間について

5.この試験の参加予定者数

6.臨床試験終了後の対応について

7.期待される効果について

8.予想される合併症と危険性について

9.この試験中に、あなたの健康に被害が生じた場合について

10.遺伝子解析などの情報の開示と期待される利益および予想される不利益について

11.この試験に関わる費用や謝礼について

12.この他の治療について

13.同意しない場合でも不利益は受けないこと

14.同意後の撤回について

15.試料等の保存及び使用方法並びに保存期間

16.治療計画書等の開示について

17.プライバシーの保護について

18.カルテなどの閲覧について

19.知的財産権の帰属について

20.当該研究に係る資金源、利益相反について

21.あなたに守っていただきたいこと

22.問い合わせ窓口

23.研究機関、研究責任者について

# **19．研究対象者の費用負担**

**19.1 健康被害の補償**

本臨床研究は、すでに厚生労働省により認められた薬剤および医療機器を使用する。したがって、本臨床研究に係る治療が原因で健康被害が生じた際は、本臨床研究の研究責任者もしくは研究分担者が責任をもって治療にあたるため、それに対する金銭的補償は行われない。何らかの障害が起きた場合にはすみやかに適切な処置と治療をもって対応するが、その際に発生する治療費は、通常の保険診療によるため研究対象者の自己負担となる。

**19.2費用負担の概要**

本研究で用いる検査および医薬品の投与は通常の保険診療内で行われるため、研究に参加することによる研究対象者の費用負担は発生しない。

# **20．記録文書等の保存**

研究責任者は、「神戸大学大学院医学研究科等における研究データ等の保存期間等に関するガイドライン」に基づき、研究実施に係わる重要な文書（倫理審査委員会への申請書類の控え、研究機関の長からの通知文書、各種申請書・報告書の控、情報公開文書、その他、データ修正履歴、ノートへの記載など研究に用いられる情報の裏付けとなる資料または記録等）を、研究の中止または終了後10年間、論文等の研究結果の公表日から10年間のいずれか遅い期日まで施錠可能な場所で保存し、その後は個人を特定できない状態にして廃棄する。

本研究に係る試料（標本）は、本研究の結果の最終の公表について報告された日から5年間(原則)保存し、その後は個人を特定できない状態にして廃棄する。

# **21．研究計画の登録**

本試験は医学雑誌編集者会議国際委員会（International Committee of Medical Journal Editors :ICMJE）の勧告に基づき、大学病院医療情報ネットワーク臨床試験登録システム（University hospital Medical Information Network-Clinical Trial Registry: UMIN-CTR）へ研究概要の登録を行う（登録番号：000032599）。研究計画書変更など、研究進捗に応じて適宜更新する。

# **22．研究資金源および各研究者のCOI状態の開示**

**22.1研究の資金源**

　本研究は保険診療適応内で行うため、研究の際に行う検査や診療自体に特に資金を必要としない。学会発表の費用等は、神戸大学大学院医学研究科内科学講座呼吸器内科学分野の研究費より負担する。

**22.2研究に関する利益相反**

　本研究に関して開示すべき利益相反はない。

# **23．研究成果の帰属と研究結果の公表**

本試験により得られた結果やデータ、知的財産権は、神戸大学医学部附属病院、呼吸器内科に帰属する。具体的な取扱いや配分は協議して決定する。

本試験の結果は、学会にて発表の後、英文専門誌に論文として投稿する。国内での学会での発表も必要に応じて行うこととする。原則として、研究結果の主たる公表論文の著者は筆頭を研究事務局とし、以下、研究責任者の順とする。それ以下は、論文の投稿規定による制限に従って共著者を選ぶ。

すべての共著者は投稿前に論文内容をreview し、発表内容に合意した者のみとする。内容に関して、議論にても合意が得られない場合、研究責任者は研究責任者の了承の上で、その研究者を共著者に含めないことができる。学会発表は複数回に及ぶ可能性があるため、研究事務局、研究責任者が指名した者の中から、持ち回りで発表を行うこととする。

# **24．研究組織**

**24.1研究機関**

１．研究責任者および研究分担者

（氏名） （診療科） （連絡先）

○立原　素子 医学研究科内科学講座呼吸器内科学分野 078-382-5660 （内線）71211

西村 善博 呼吸器内科 同上 （内線）71200

小林　和幸 医療の質・安全管理部 同上 (内線) 71203

山本　正嗣 呼吸器内科 同上 (内線) 71201

桂田　直子 医学研究科内科学講座呼吸器内科学分野 同上 (内線) 71202

神保　直江 病理診断科 078-382-6474 (内線)74812

桐生　辰徳 同 (内線)71216

安田　裕一郎 同 (内線)71220

大歳　丈博 同 (内線)71208

吉崎　飛鳥 同　 (内線)71213

　 小山　貴与子 同 (内線)71217

湯村　真沙子 同 (内線)71218

古川　皓一 同 　　　　　　(内線)71219

　 吉岡　潤哉 同　　　　　　　　　　　　　　　　　　　　　　　　　　　　　　 (内線)71221

三村　千尋　　　　　呼吸器内科　　　　　　　　　　　　　　　　　　　　　 同上　　　　　　　 　　(内線) 71204

佐藤 宏紀　　　　　同　　　　　　　　　　　　　　　　　　　　　　　　　　　　同上　　　　　　　　　 (内線) 71205

髙田 尚哉 同　　　　　　　　　　　　　　　　　　 　　　　　　　　 同上　　　　　　　　　(内線) 71209

（○印：研究責任者）

２．研究事務局

桂田直子 神戸大学大学院医学研究科　内科学講座・呼吸器内科学分野　特命助教

〒650-0017 神戸市中央区楠町7-5-2

TEL：078-382-5660

FAX：078-382-5661

E-mail： nk1208@med.kobe-u.ac.jp

3．データ管理責任者

桂田直子 神戸大学大学院医学研究科　内科学講座・呼吸器内科学分野　特命助教

4．モニタリング責任者

永野　達也　　　医学研究科内科学講座呼吸器内科学分野　 特命助教

5．監査責任者

なし　(監査は行わない)

6．統計解析責任者

桂田直子 神戸大学大学院医学研究科　内科学講座・呼吸器内科学分野　特命助教

**24.2研究対象者等及びその関係者からの相談等への対応方法**

相談窓口の連絡先と受付時間

　　桂田直子 神戸大学大学院医学研究科　内科学講座・呼吸器内科学分野　特命助教

〒650-0017 神戸市中央区楠町7-5-2

TEL：078-382-5660

FAX：078-382-5661

E-mail： nk1208@med.kobe-u.ac.jp

受付時間：　10：00 - 17：00　（土日祝日はのぞく）

**24.3委託業務内容及び委託先**

委託業務なし

# **25．文献リスト・参考資料**

1. 国立がん研究センター　がん情報サービス　最新がん統計<https://ganjoho.jp/reg_stat/statistics/stat/summary.html>

2. 日本肺癌学会　肺癌患者におけるPD-L1 検査の手引き　第一版

https://www.haigan.gr.jp/uploads/photos/1400.pdf

3. Hayama M, Izumo T, Matsumoto Y, Chavez C, Tsuchida T, Sasada S. Complications with Endobronchial Ultrasound with a Guide Sheath for the Diagnosis of Peripheral Pulmonary Lesions. Respiration; international review of thoracic diseases. 2015; 90:129-35.

4. Minami D, Takigawa N, Morichika D, Kubo T, Ohashi K, Sato A, Hotta K, Tabata M, Tanimoto M, Kiura K. Endobronchial ultrasound-guided transbronchial biopsy with or without a guide sheath for diagnosis of lung cancer. Respiratory investigation. 2015; 53:93-7.

5. Ishida T, Asano F, Yamazaki K, Shinagawa N, Oizumi S, Moriya H, Munakata M, Nishimura M; Virtual Navigation in Japan Trial Group. Virtual bronchoscopic navigation combined with endobronchial ultrasound to diagnose small peripheral pulmonary lesions: a randomised trial. Thorax. 2011; 66:1072-7.

6. Zhang L, Wu H, Wang G. Endobronchial ultrasonography using a guide sheath technique for diagnosis of peripheral pulmonary lesions. Endoscopic ultrasound. 2017; 6:292-9.

7. Xu CH, Yuan Q, Yu LK, Wang W, Lin Y. Endobronchial ultrasound transbronchial biopsy with guide-sheath for the diagnosis of solitary pulmonary nodules. Oncotarget. 2017; 8:58272-7.

8. Chavez C, Sasada S, Izumo T, Watanabe J, Katsurada M, Matsumoto Y, Tsuchida T. Endobronchial ultrasound with a guide sheath for small malignant pulmonary nodules: a retrospective comparison between central and peripheral locations. Journal of thoracic disease. 2015; 7:596-602.

改定履歴

| 版番号 | 作成・改定日 | 改定理由／内容 |
| --- | --- | --- |
| 第1版 | 2018年5月1日 | 新規制定 |
| 第1.1版 | 2018年7月18日 | 改訂 (目標症例数の設定、主要評価項目追記) |
| 第1.2版 | 2018年9月18日 | 改訂 (選択基準の改訂、症例登録の追記) |
| 第1.3版 | 2019年3月31日 | 改訂 (研究分担者の変更) |
| 第1.4版 | 2019年6月12日 | 改訂 (目標症例数の設定、研究分担者の変更) |
| 第1.5版 | 2019年10月21日 | 改訂 (試験実施期間の延長) |
| 第1.6版 | 2020年3月31日 | 改訂 (研究分担者の変更) |
