# Supplementary material for: Yield of tumor samples with a large guide-sheath in endobronchial ultrasound transbronchial biopsy for non-small cell lung cancer: A prospective study
Source: PLoS One. 2021 Oct 29;16(10):e0259236. doi: 10.1371/journal.pone.0259236 (PMC8555788; doi:10.1371/journal.pone.0259236)
Supplement: S3 File — (DOCX) [file pone.0259236.s004.docx]

Yield of tumor samples with a large guide-sheath in endobronchial ultrasound transbronchial biopsy for non-small cell lung cancer: a prospective study

Version 1.6 March 31, 2020

| Principal investigator |
| --- |
| Name: Motoko Tachihara  Division of Respiratory Medicine, Department of Internal Medicine, Kobe University Graduate School of Medicine TEL: +81-78-382-5660  FAX: +81-78-382-5661  E-mail: mt0318@med.kobe-u.ac.jp |

| Study Secretariat |
| --- |
| Name: Naoko Katsurada  Office Address: 7-5-2 Kusunomachi, Chuo-ku, Kobe  Tel: +81-78-382-5660  FAX: +81-78-382-5661  E-mail: nk1208@med.kobe-u.ac.jp |

Table of contents

[**1．**Overview of the study 3](#_Toc514002070)

[**2．**Background and Purpose 4](#_Toc514002071)

[**3．**Outline of the study drug or device 7](#_Toc514002072)

[**4．**Research Subjects 7](#_Toc514002073)

[**5．**Study Method 8](#_Toc514002074)

[**6．**Evaluation items 12](#_Toc514002075)

[**7．**Observations and Examination items 13](#_Toc514002076)

[**8．**Discontinuation Criteria 15](#_Toc514002077)

[**9．**Handling of adverse events 16](#_Toc514002078)

[**10．**Discontinuation, suspension or termination of the test 18](#_Toc514002079)

[**11．**Study period 18](#_Toc514002080)

[**12．**Subjects of analysis and statistical analysis methods (main and secondary analyses) 18](#_Toc514002081)

[**13．**Changes to the study protocol, case report form or analysis plan 21](#_Toc514002082)

[**14．**Data Management 21](#_Toc514002083)

[**15．**Quality Assurance for Clinical Trials 22](#_Toc514002084)

[**16．**Correspondence to the Ethical Guidelines and Declaration of Helsinki 22](#_Toc514002085)

[**17．**Consideration for the human rights and safety and disadvantage of research subjects 22](#_Toc514002086)

[**18．**Approach to explain to research subjects and obtain their consent (informed consent) 24](#_Toc514002087)

[**19．**The cost of the research subjects 26](#_Toc514002088)

[**20．**Retention of documents and records 26](#_Toc514002089)

[**21．**Registration of the research plan 26](#_Toc514002090)

[**22．**Disclosure of research funding sources and COI status of each researcher 27](#_Toc514002091)

[**23．**Attribution of Research Results and Publication of Research Results 27](#_Toc514002092)

[**24．**Research Organization 27](#_Toc514002093)

[**25．**Bibliography and Reference Materials 29](#_Toc514002094)

**1．Overview of the study**

**1.1** Title

Yield of tumor samples with a large guide-sheath in endobronchial ultrasound transbronchial biopsy for non-small cell lung cancer: a prospective study

**1.2** Purpose

We investigate whether there is a difference in the number of tumor cells in specimens obtained by bronchoscopy of peripheral lung lesions suspected of lung cancer between a small-diameter guide sheath (GS) and a large-diameter GS by comparison between a prospective registry (all cases biopsied with a large-diameter GS) and a retrospective registry (cases previously biopsied with a small-diameter GS).

Primary endpoint

The number of tumor cells of specimens obtained by transbronchial biopsy (TBB) with large GS (prospectively enrolled patients) and those obtained by TBB with small GS (retrospectively enrolled patients) .

Secondary endpoint

1. Evaluate the following items of the prospective and retrospective registration groups

a. The lesion is visualized by ultrasound or not.

b. The success rate of tissue diagnosis

c. The number of samples from which tumor tissue was obtained in all samples

d. Sample size

e. The success rate of PD-L1 testing

f. The proportion of tumor cells in the specimen's nucleated cells

　 g. Complications

2. Only the prospective registration group will be assessed on the following items

The rate of change to a small-diameter GS
Tumor size, location, and the character of the lesion, and involved bronchi of lesions that did not required to change to the thinner bronchoscope and those that needed this change.

**1.3** Subject

Patients who meet all of the following eligibility criteria and do not meet any of the following exclusion criteria will be eligible for this study.

1.3.1 Inclusion criteria:

1. Patients aged 20 years or older at the time of obtaining consent.

2. patients who undergo bronchoscopy using endobronchial ultrasonography (EBUS) with GS
for peripheral lung lesions suspected to be non-small cell lung cancer (NSCLC)

3. The written consent to the study has been obtained.

1.3.2 Exclusion criteria:

1. Patients with visible lesions by bronchoscopy.

2. Patients underwent re-biopsy after treatment of lung cancer.

3. Patients with lesions suspected to be benign on CT or other imaging studies.

4. Patients with serious complications that may interfere with the examination.

5. Patients who are taking anticoagulants or antiplatelet agents and cannot discontinue them temporarily (except when temporary change to heparin is possible).

6. Pregnant women.

7. Patients judged to be unsuited for this study by their physicians.

**1.4** Number of cases

Expected registrations: 80 prospective registrations

　　　 New enrollment will be terminated when 36 NSCLC cases are obtained by large-diameter GS.

Retrospective registration: 36 cases are assumed; (equal to the number of NSCLC cases obtained with large-diameter GS among the prospective registrants)

Registration Period:

Prospective Registration: Ethics Review Board Approval Date (July 24, 2018) – December 31, 2020

Retrospective registration: January 1, 2017 – December 31, 2017

Analysis period: January 1, 2021 – June 30, 2021

**1.5** Contact information

Name: Naoko Katsurada

Affiliation: Department of Respiratory Medicine, Kobe University Hospital

The address of the research office is 7-5-2 Kusunomachi, Chuo-ku, Kobe City, Hyogo Prefecture

TEL: +81-78-382-5660, FAX: +81-78-382-5661

E-mail: nk1208@med.kobe-u.ac.jp

2．Background and Purpose

**2.1** Background

In Japan, the number of patients with and deaths from lung cancer have been increasing, making it the leading cause of cancer deaths^1)^, and the importance of lung cancer treatment has been increasing. At the same time, new drugs that target genetic mutations such as EGFR and immune checkpoint inhibitors have been introduced one after another, and patients who are eligible for such drugs are experiencing a dramatic increase in survival. Lung cancer practice guidelines recommend selection of therapeutic agents in accordance with genetic mutations in tumor tissue and programmed death ligand 1 (PD-L1) results, increasing the importance of tumor tissue sampling. Pembrolizumab, an immune checkpoint inhibitor, requires tissue confirmation of PD-L1 positivity prior to administration, and more than 100 viable tumor cells are required in tissue for this evaluation^2)^ and sufficient tumor volume is required.

So far, bronchoscopy has been devised in various ways to increase the tissue diagnosis rate. The ultrasound probe with guide sheath is inserted through the bronchoscope forceps channel to the vicinity of the lesion to confirm the position of the lesion with echo, and biopsy and abrasion are performed by inserting biopsy forceps and cytology brushes into the guide sheath placed at the same site. endobronchial ultrasonography with a guide sheath (EBUS-GS) has been developed and shown to have a good diagnostic rate and safety^3,4)^. Virtual bronchoscopic navigation (VBN), which uses virtual images of the route to the lesion using CT images taken beforehand to guide the bronchoscope and instruments, has improved the diagnostic rate^5)^. Biopsy with EBUS-GS is a well-established technique with a diagnostic rate of 60-80% for peripheral lung lesions^6)^ and has been introduced at many institutions that treat lung cancer.

Biopsy forceps that can currently be used in the EBUS-GS procedure to obtain tissue are available as a disposable guide sheath kit from Olympus Medical, Inc. in the form of K-202 (forceps OD 1.5 mm, applicable channel diameter 2.0 mm, referred to as "Small-diameter GS") and K -203 (forceps outer diameter 1.9mm, 2.6mm channel diameter, referred to as "Large-diameter GS"); forceps used in the Large-diameter GS kit allow for larger specimens than those used in the Small-diameter GS. The small-diameter GS is for a bronchoscope with a channel diameter of 2.0mm (Olympus Medical P290 (4.2mm), Q290 (4.8mm outer diameter), etc.) and the large-diameter GS is for a bronchoscope with a channel diameter of 2.8-3.0mm (Olympus Medical 1T260 (5.9mm outer diameter), 1TQ290 (5.9 mm outer diameter), etc.). Although large-diameter GSs have the advantage of obtaining large specimens, they have the disadvantage of making it difficult to select the involved bronchi, especially in the upper periphery of the upper lobe, when compared to thin-diameter bronchoscopes. Currently, in Japan, small-diameter GS is frequently used, and even in institutions that actively use large-diameter GS, there are no clear criteria for selection of GS, leaving it up to the judgment of the operator to decide which one to use. In a study in which all patients with pulmonary nodules (tumor diameter of 3 cm or less) were biopsied with large-diameter GS, a diagnostic rate of 74.4% (83.3% in malignant tumors only) was reported^7)^, which was comparable to that of previous studies using small-diameter GS. In a retrospective study of the diagnostic rate of EBUS-GS with VBN for pulmonary nodule shadows, the choice of GS was determined at the discretion of the operator, but it was reported that there was no difference in the diagnostic rate due to differences in GS diameter in a sub-analysis of the study^8)^. Although the number of reports is small and not directly comparable, the diagnostic rate of large-diameter GSs is thought to be comparable to that of small-diameter GSs, the number of tumor cells in the obtained specimens has not been examined to date. It is necessary to obtain large specimens to perform molecular analysis targeting multiple genes and the evaluation of the PD-L1 expression. In fact, our department has been using a small-diameter GS, but in about 3% of the cases submitted for PD-L1 examination, PD-L1 was enable to evaluated because of inadequate tumor samples, and in some cases, bronchoscopy was performed again due to low tumor cell counts.

**2.2** Purpose of the study

2.2.1 Primary purpose

To evaluate the feasibility of collecting a larger number of tumor cells using a large-diameter GS for bronchoscopy of peripheral lung lesions suspected being lung cancer.

2.2.2 Secondary purposes

a. Evaluate the rate of lesion delineation for large-diameter and small-diameter GS. (The numerator is the case in which the lesion could be visualized by ultrasound, and the denominator is all cases of large-diameter and small-diameter GS, respectively.)

b. Evaluate the histopathological diagnosis rates of large-diameter and small-diameter GS.(The numerator is the case in which the histological diagnosis was possible, and the denominator is all cases of thick and fine GS, respectively.)

c. Evaluate the number of specimens for which tumor tissue was obtained from all samples obtained from large-diameter and small-diameter GS.

d. Evaluate the size of the specimens of large-diameter and small-diameter GS

e. Evaluate the success rate of PD-L1 testing in large-diameter and small-diameter GS.

f .Evaluate the proportion of tumor cells in nucleated cells in samples of large and small diameter GS.

g. Evaluate the difference in complications such as hemorrhage and pneumothorax between cases with large-diameter and small-diameter GS.

2. Evaluate the rate of change to small-diameter GS, size, location, character, and involved bronchi. Clinical significance: Identifying lesions that are difficult to diagnose with large-diameter GS facilitates the selection of the appropriate type of GS.

2.2.3 Purpose of the exploration

Not applicable.

# **3．Outline of the study drug or device**

**3.1** Study Equipment Information

Olympus Medical's Disposable Guide Sheath Kit

|  | K-202 (Small-diameter GS) | K-203 (Large-diameter GS) |
| --- | --- | --- |
| guide sheath | SG-200C | SG-201C |
| outer diameter | 1.95mm | 2.55mm |
| biopsy forceps | FB-233D | FB-231D |
| outer diameter | 1.5mm | 1.9mm |
| cytology brush |  | BC-202D-2010 |
| outer diameter |  | 1.8mm |

Instructions for use：The guide sheath is inserted through the bronchoscope's forceps channel to the vicinity of the lesion, and the location of the lesion is confirmed by EBUS, and biopsies and brushing are performed by inserting instruments with biopsy forceps and cytology brushes into the guide sheath inserted at the same site. The K-202 kit does not contain a cytology brush, so uses the BC-202D-2010.

Both kits are covered by Japanese health insurance.

**3.2** Expected Failure

Although these devices have been widely used since they were placed on the market and their safety is assured, there is a rare possibility of damage to the device, degradation of function such as a biopsy forceps not opening, misalignment of the radiopaque tip of the guide sheath, and loss of the device.

# **4．Research Subjects**

Patients who meet all of the following inclusion criteria and do not meet any of the following exclusion criteria will be considered eligible for study in this study.

**4.1** Inclusion criteria:

Patients who meet all of the following criteria will be eligible

1. Patients aged 20 years or older at the time of obtaining consent.

2. patients who undergo bronchoscopy using endobronchial ultrasonography (EBUS) with GS
for peripheral lung lesions suspected to be NSCLC

3. The written consent to the study has been obtained

Reason for inclusion criteria:

1. The age at which personal consent is legally established.

2. It was set up to identify the subject of this study.

3. Established for ethical considerations.

**4.2** Exclusion Criteria:

Patients with any one of the following will be excluded

1. Patients with visible lesions by bronchoscopy.

2. Patients underwent re-biopsy after treatment of lung cancer.

3. Patients with lesions suspected to be benign on CT or other imaging studies.

4. Patients with serious complications that may interfere with the examination.

5. Patients who are taking anticoagulants or antiplatelet agents and cannot discontinue them temporarily (except when temporary change to heparin is possible).

6. Pregnant women.

7. Patients judged to be unsuited for this study by their physicians.

Reason for exclusion criteria:

(1)(2)(3) This was set because of the potential impact on the evaluation of effectiveness.

(4)(5)(6) In order to maintain the safety of the study, patients who were unsuitable for inclusion in this study were excluded.

(7) To leave a possibility for the person in charge to consider.

# **5．**Study Method

**5.1** Type and design of the study

　　Prospective intervention trials (historical cohort analysis)

　　　The results of this prospective intervention trial and the result of historical cohort analysis of the small-diameter GS will be performed.

　　Rationale: We used only small-diameter GS until December 2017. In the prospective target group, it is reasonable to use the previous consecutive cases of small-diameter GS use as a historical control.

**5.2** Study outline (see study flow chart)

This is a single-center, historical controlled study to evaluate the benefit of large-diameter GS in patients with peripheral lung lesions.

All 80 study subjects (prospective enrollment group) undergoing TBB for peripheral pulmonary lesions will be performed with a large-diameter GS. When the lesion was invisible in EBUS, we will change to a thinner bronchoscope and TBB was performed with a small GS. Furthermore, we will enroll consecutive patients with NSCLC in whom tissue samples were previously obtained by EBUS-GS TBB using a small GS kit from January 1 to December 31, 2017 (assumed to be 36 cases, retrospective registration group). The primary endpoint of the study will be the difference in the number of tumor cells obtained between the prospective and retrospective groups. The study period will consist of three days: the day before the bronchoscopy, the day of the bronchoscopy, and the day the bronchoscopy results are explained. A summary of the study schedule is shown in Figure 1.

BF

confirm registration

Pathological diagnosis

Primary Registration

consent acquisition

・Group of prospective registration

Screening

test

Pathology specimen evaluation

（count of tumor cells）

・Group of retrospective registration

Pathology specimen evaluation

（count of tumor cells）

・後ろ向き登録群

Registration

**5.3** Case enrollment and allocation methods

The principal investigator or research co-investigator will follow the following procedures from obtaining consent from the research subjects to the start of the study.

1. After obtaining consent from research subjects, the principal investigator or research co-investigator will conduct a screening test to determine the eligibility of research subjects. If the research subject's consent is obtained, the test results before the consent is obtained can be used.
2. The principal investigator or co-investigator, after confirming that the subject meets all inclusion criteria and none of the exclusion criteria, completes the "Case Registration Form". To facilitate the identification of research subjects, a list of research subject identification numbers will be created.
3. The research secretariat will confirm eligibility and issue a Confirmation of Case Registration Form with the decision result and registration number. (Primary Registration)
4. The principal investigator or research co-investigator will confirm the decision on the Confirmation of Case Enrollment Form and initiate the study treatment. Study examination must not be started until the written confirmation of case enrollment is received. Secondary enrollment (confirmatory registration) will be conducted after bronchoscopy and observation of the lumen.
5. The Case Registration Form and the Confirmation of Case Registration Form shall be properly retained as source documents.

| consent acquisition | ⇨ | screening test | ⇨ | Eligibility Verification | ⇨ | Completing the Case Registration Form | ⇨ | Primary Case  Registry | ⇨ | Receiving confirmation of case registration | ⇨ | Screening test and  Registration | ⇨ | Study begins |
| --- | --- | --- | --- | --- | --- | --- | --- | --- | --- | --- | --- | --- | --- | --- |

**5.4** Examination procedure

EBUS TBB with large-diameter GS is performed for peripheral lung lesions. If it is difficult to visualize the lesion with a large-diameter GS, change to a small-diameter GS for specimen collection.

1. Before the examination

Virtual Image Creation

CT reconstruction data (in DICOM format) is loaded into Bf-NAVI (Olympus Medical Systems) to create a virtual image to the target. If the route to the involved bronchus can be easily determined, Bf-NAVI is not necessarily required.

Identify the size, location, presence or absence of involved bronchi, and characteristics of the tumor to be targeted on CT.

If the lesion is a very high probability of small cell lung cancer, registration will be excluded.

2. Examination

1) Pretreatment

After pharyngeal anesthesia with 4% xylocaine viscus, 4% xylocaine is sprayed with Jackson's spray.

Administer hydroxyzine hydrochloride 25 mg intramuscularly, or intermittent IV midazolam, as appropriate.

2) Bronchoscope insertion, surface anesthesia, endoscopic observation

A bronchoscope (Olympus Medical Systems, 1TQ290, 1T260) is inserted, and 2% xylocaine is used to anaesthetize the bifurcation and other bifurcations. 2 ml of 2% xylocaine is sprayed twice at each bifurcation, and excess xylocaine and secretions are immediately aspirated. After superficial anesthesia is administered to each regional branch, the bronchial lumen is observed. Confirm that there is no obvious tumor outcrop or responsible bronchial stenosis, and secondary enrollment (confirm enrollment) is made. If not enrolled, continue bronchoscopy as a routine practice without enrolling in the study.

3) Identification of the lesion and GS implantation

A bronchoscope is inserted into the involved bronchus according to the information of the VBN image or CT imaging information, and an ultrasound probe with a large-diameter GS (SG-201C) is inserted into the forceps, and the lesion is confirmed by EBUS while confirming the location of the lesion under X-ray fluoroscopy. A 20 MHz mechanical radial scanning probe (XUM-S20-20R) with an outer diameter of 2.0 mm is used for the large-diameter GS ultrasound probe (all manufactured by Olympus Medical Systems). If the lesion cannot be delineated after insertion of the large-diameter GS, a curette-type inductor is used or the lesion is changed to a small-diameter GS (SG-200C) for delineation. When changing to a small-diameter GS, change the bronchoscope to a small-diameter bronchoscope (P290, or P260F) and confirm the lesion with EBUS using a narrow-diameter GS ultrasound probe (XUM-S20-17S, 1.7 mm in outer diameter). After the lesion is delineated, the GS is left in the lesion and the ultrasound probe is removed.

4) Biopsy

Biopsy forceps are inserted into the GS and the lesion is examined by forceps and brush cytology. Five biopsies are taken. Brush-bleed cytology is performed twice, alternating biopsies and brushes. Thereafter, additional specimen collection with a TBNA needle, curettes, and usually forceps is performed as needed.

When withdrawing the GS, a 20-second sustained aspiration with a 20 ml syringe is performed with the GS lodged at the lesion site, and then the GS is withdrawn.

3. After examination (specimen processing)

Tissue biopsy specimens are subjected to hematoxylin and eosin staining and, if necessary, special immunostaining to make a pathological diagnosis. Tumor cell counts are measured by one pathologist and one cytologist. The abrasion cytology specimen is stained with Papanicolaou stain and cytological diagnosis is made. Biopsy forceps and cells attached to the abrasion brush are washed with saline, and cytology, general bacterial and antacid smears, and culture tests are performed. In addition, specimens aspirated through the GS are mixed with device washing saline to create a cell block, if necessary.

**5.5** Provisions for concomitant medication (therapy)

not applicable

**5.6** Methods of withdrawal

not applicable

**5.7** Management and delivery procedures for study equipment

No special management is required for the study as it is used for bronchoscopy of peripheral pulmonary lesions within health insurance.

**5.8** Medication guidance information

not applicable

**5.9** Post-treatment

not applicable

**5.10** Post-study follow up

In cases that do not lead to a definitive diagnosis, we recommend repeat bronchoscopy or other tests that lead to a definitive diagnosis (CT-guided lung biopsy or thoracoscopic lung biopsy). In the event that the patient did not require further diagnostic procedures, the lesion was followed-up for 2 years. The final diagnosis was based on pathological evaluation or clinical follow-up. If there is no enlargement, the patient should be considered non-neoplastic.

**6．Evaluation items**

**6.1** Primary endpoint

The number of tumor cells of specimens obtained by transbronchial biopsy (TBB) with large GS (prospectively enrolled patients) and those obtained by TBB with small GS (retrospectively enrolled patients) .

**6.2** Secondary endpoint

6.2.1 Efficacy endpoints

　Evaluate the following items for prospective and retrospective registration groups

(1) The lesion is visualized by ultrasound or not.

　　(Evaluate whether ultrasound was able to delineate the lesion.)

(2) The success rate of tissue diagnosis

　　(Assess whether a tissue diagnosis could be obtained.)

(3) The number of samples from which tumor tissue was obtained in all samples

　　(Evaluate the number of specimens from which tumor tissue could be obtained out of all specimens.)

(4) Sample size

(5) The success rate of PD-L1 testing

(6) The proportion of tumor cells in the specimen's nucleated cells

6.2.2 Safety assessment

complications

　　 Bleeding, pneumothorax, infection and other complications will be evaluated in both groups.

6.2.3 Only the prospective registration group will be assessed on the following endpoints

・The rate of change to a small-diameter GS

・Tumor size, location, and the character of the lesion, and involved bronchi of lesions that did not required to change to the thinner bronchoscope and those that needed this change.

# **7****．Observations and Examination items**

**7.1** Implementation Schedule and Procedures

The schedule for observation, examination, and evaluation is shown in the following table. The principal investigator or researcher will carry out observations, inspections, etc. according to the schedule.

| Date |  | Before bronchoscopy  (Screening test)  Visit 1 | under examination  Visit 2 | After Examination  (At the time of finding pathology.)  Visit 3 |
| --- | --- | --- | --- | --- |
| Item | Chest radiographs  Blood tests (blood count, biochemistry, coagulation and infection)  EKG  Respiratory function test  Chest CT  Bronchoscopy  Pathological examination | 〇  〇  〇  〇  〇 | 〇 | 〇 |
| observation items | Background of the Study Subjects  vital signs  Tumor size and location  Number of branches that can be inserted  Change to small GS  Lesion delineation  Number of biopsies  Number of samples  Complications  Number of tumor cells in the specimen  Tissue diagnosis  Size of specimens  Number of species  Evaluability of PD-L1 test  proportion of tumor cells in specimens | 〇  〇  〇 | 〇  〇  〇  〇  〇  〇  〇 | 〇  　　　　　　〇  〇  〇  〇  〇  〇 |

Data related to this study may be used before consent is obtained, but will be used only after obtaining the research subject's consent.

7.1.1 Screening test

The principal investigator or research co-investigator will conduct the following screening tests, and patients who meet the selection criteria and do not conflict with the exclusion criteria will be eligible for study. The tests will be as described below.

1. Background of the study population (current medical history, medical history, comorbidities, prescription drugs, allergies)

2. Vital signs (heart rate, blood pressure, temperature, SpO_2_)

3. Hematology (WBC, RBC, Hb, Hct, Plt, white blood cell fractionation), coagulation system (PT, APTT)

4. Biochemical tests (AST, ALT, T-BIL, TP, ALB, BUN, CRP, Na, K, Cl, Cr)

5. EKG.

6. Chest radiographs

7. Respiratory function test

7.1.2 Information on Research Subjects

At the time of consent acquisition or screening, the following research subject information will be recorded.

1. Date of obtaining consent

2. Subject identification code

3. Background of the research subjects

Age at the time of obtaining consent

Height/ Weight

Prescription drugs

7.1.3 Observations, examination and evaluation items

The test items for each visit are listed below.

**Visit 1** Before bronchoscopy

(This will be done on the same day as the screening, as it is performed as a pre-bronchoscopy examination in routine clinical practice.)

1. Chest CT

Tumor size

Tumor location

Presence or absence of involved bronchi.

Characteristics of tumor

**Visit 2** The day of the bronchoscopy

1. Bronchoscopy

　　　　　The number of bronchial branches can be observed by bronchoscopy

　　　　　Characteristics of large EBUS findings

　　　　　The rate of change to small-diameter GS

　　　　 If the GS is changed to a small-diameter GS, the EBUS findings

Number of biopsies

　　　　　Number of specimens

　　　　　Antibacterial drug for prevention

　　　　　Complications

**Visit 3** When explaining the results of a bronchoscopy

1. Complications

**(**No Visit**)** Pathological examnation

1. Tumor cell count

2.　Sample size

3. Diagnosis of the tissue

4. Evaluability of PD-L1 testing

5.　Proportion of tumor cells in nucleated cells.

**7.2** Provision of samples and information to other organizations

No provision will be made.

**7.3** Handling of Deviations from the Implementation Plan

The principal investigator or research co-investigator will not make any deviations or changes from the study protocol prior to obtaining approval from the head of the institution based on prior review by the Research Ethics Review Committee.

The principal investigators or research contributors may deviate or modify the study protocol for unavoidable reasons such as emergency avoidance, etc., prior to obtaining the prior approval of the research ethics review committee. In such cases, the principal investigators or research contributors will promptly submit the details of and reasons for the deviation or change and the proposed revision of the study protocol or other documents, if necessary, to the Research Ethics Review Committee for approval by the Research Ethics Review Committee and the head of the research institution.

The principal investigator or research co-investigator shall record all deviations from the study protocol, including the reasons for the deviation.

If the principal investigator or research co-investigator learns that the study does not conform to the ethical guidelines for medical research involving human subjects (only when the degree of non-conformity is significant), the principal investigator or research co-investigator must promptly report to the head of the institution, and cooperate with the head of the institution in reporting and disclosing the status and results of the response. After taking the necessary action, the principal investigator or research participant shall cooperate with the head of the research institution in reporting to the Minister of Health, Labor and Welfare or other relevant authorities on the status and results of such action.

# **8****．discontinuance criteria**

If the principal investigator or research co-investigator determines that the study cannot be continued for any of the following reasons, the principal investigator or research co-investigator must stop the study, and document the date and time of discontinuation/withdrawal, the reason for discontinuation/withdrawal, and the course of the study in the medical record and the CRF, as well as performing the necessary tests at the time of discontinuation/withdrawal to evaluate the efficacy and safety.

1. If a research subject declines to participate in the study or withdraws his or her consent

2. If it is found after registration that the eligibility is not satisfied

3. When it is difficult to continue the test due to adverse events

4. If the entire study is cancelled

5. When the principal investigator or research co-investigator determines that it is appropriate to discontinue the study for other reasons.

# **9．Handling of adverse events**

**9.1** Definition of Adverse Events

Adverse events are any unfavorable or unintended signs (including abnormal changes in laboratory values), symptoms, or diseases that occur in conjunction with bronchoscopy, regardless of their causal relationship to the test.

**9.2** Response to Research Subjects in the Event of an Adverse Event

In the event of an adverse event, the principal investigator or research co-investigator participant will immediately take appropriate action and record it without any discrepancy in the medical record and case report form. In the case of discontinuation of the test or treatment for the adverse event, the researcher will inform the subject of the event.

**9.3** Adverse Events to be Reported

All adverse events occurring from the start to the end of the test, whether or not they are causally related to the test, should be reported and observed until the adverse events resolve or are fixed. In addition, all adverse events determined to be causally related to the test should be reported until the end of the study period.

**9.4** Reporting Procedures in the Event of an Adverse Event

All adverse events occurring during the above period will be documented by the principal investigator or research co-investigator without any discrepancies in the medical records and case report forms.

**9.5** Information required for the assessment of adverse events

The severity of an adverse event is defined as: (1) mild: when the patient gets better without treatment by stopping the test alone; (2) moderate: when the test is stopped and some treatment is required, but not severe; and (3) severe: when the test is stopped and the patient needs to be hospitalized or is life-threatening.

・Name of the adverse event

・Name of the adverse event

・Date of conversion

・Outcome: recovery, lightening, with sequelae, no recovery, death, unknown

・Treatment (administration of study drug): no change, discontinuation, withdrawal, reduction, increase in dose, not applicable・Severity: mild, moderate, severe

・Causal relationship with study drug: relevant, not relevant

**9.5.1** Causal relationship between the resilience of adverse events and the study drug

Recovery of an adverse event is defined as the absence of an adverse event or an improvement in status prior to administration. Determination of test causation in adverse events will be based on consideration of the study subject's general condition, comorbidities, concomitant medications/conventional therapies, and time relationship.

**9.6** Handling in the Event of a Serious Adverse Event

**9.6.1** Definition of Serious Adverse Events

Serious adverse events shall be defined as any of the following

1. Death

2. Items that may lead to death

3. Disability (development of dysfunction to the extent that it interferes with daily life)

4. Items that may lead to impairment

5. The patient needs to be hospitalized or extended in the hospital or clinic for treatment.

6. Serious condition according to the cases listed in (1) to (5) above.

**9.6.2** Serious adverse events to be reported

Report all serious adverse events during the study period and any serious adverse events suspected to be related to bronchoscopy after the end of the study (discontinuation).

**9.6.3** Serious Adverse Event Reporting Procedure

When an adverse event occurs and is judged by the principal investigator or others to be serious, the adverse event information will be handled in accordance with the following procedures

1. Report from the principal investigator to the head of the institution and the principal investigator

The principal investigator will report the adverse event information to the head of the host institution and the principal investigator as soon as possible, regardless of the causal relationship. The first report (emergency report) and the second report (detailed report).

2. Report to the Minister of Health, Labour and Welfare

In accordance with the Ethical Guidelines for Medical Research Involving Human Subjects, when the head of an institution determines that a report to the Minister of Health, Labour and Welfare is necessary, the head of the institution shall prepare a "Report of Unexpected Serious Adverse Events" (in accordance with the attached form designated by the Ministry of Health, Labour and Welfare) and report it to the Minister of Health, Labour and Welfare.

3. Handling when additional information is available

when additional information on the event is obtained, the principal investigator of the institution where the adverse event occurred shall submit a report to the head of the institution as soon as possible. The handling of such additional information shall be in accordance with the procedures of (1) and (2).

4. Handlings based on the Drug and Medical Device Safety Information Reporting System

Post-marketing drugs and medical devices will be handled in accordance with the drug and medical device safety information reporting system and reported to the Ministry of Health, Labour and Welfare when necessary.

**10．Discontinuation, suspension or termination of the test.**

# **10.1** Criteria for stopping or suspending the entire study

The Research Office will consult with the principal investigator on the suspension or discontinuation of the entire study when the following information is available and it is considered difficult to continue the entire study

1.Occurrence of unanticipated serious adverse events

2. Information indicating that the trend of occurrence, such as the number, frequency, and conditions of serious adverse events that can be expected cannot be predicted from the package insert.

3.Information on serious adverse events that have been judged to have no causal relationship, but have since been judged to have an undeniable causal relationship based on the number of occurrences, frequency of occurrences, conditions of occurrence and other trends

4. Reports of studies showing that there is a risk of serious disability or death

5. Information suggesting that the study was not validated

**10.2** Information suggesting that the study was not validated

In the event of stopping or suspending the entire study, the principal investigator will promptly notify the head of the research institution in writing, detailing the reason for the stoppage or suspension. They will also promptly inform the research subjects who are receiving the study and take appropriate action, such as changing to appropriate treatment.

**10.3** Procedures for stopping or suspending a study at an individual site

In the event of the suspension or interruption of a study, the principal investigator shall promptly notify the head of the institution in writing and provide a detailed written explanation of the suspension or interruption.

**10.4** Completion of the study

Upon completion of the study, the principal investigator shall notify the head of the institution in writing that the study has been completed and provide a written summary of the study results.

# **11．Study period**

Prospective Case Registry

Ethics Review Committee approval date (July 24, 2018) – December 31, 2020

Retrospective case registration

January 1, 2017 – December 31, 2017

Analysis period: January 1, 2021 – June 30, 2021

**12．Subjects of analysis and statistical analysis methods (main and secondary analyses)**

The following is a summary of the statistical analysis plan for this study.

The statistical analysis plan may revise the summary of this study protocol; however, the study protocol will be revised if the definitions of the primary endpoints and methods of analysis change.

**12.1** Analysis target population

12.1.1 Full analysis set (FAS)

The maximum analytic population (FAS) will be all study subjects who are enrolled in the study, who have undergone bronchoscopy and for whom efficacy data are available. However, subjects in serious violation of the study protocol (e.g., failure to obtain consent, out-of-date enrollment) will be excluded.

12.1.2 Target population (per protocol set (PPS)) in compliance with the study protocol

Study subjects will be eligible for the study, except for cases in which the FAS has found the following serious violations of the study protocol's regulations, including the study methodology

Violation of the inclusion criteria

Violation of the exclusion criteria

12.1.3 Population for Safety Analysis

Not applicable.

**12.2** Target number of cases and rationale for setting up

Targeted number of cases: Large-diameter GS (prospectively enrolled): 80

New enrollment will be closed when 36 NSCLC cases are obtained by TBB with large-diameter GS.

Assume 36 NSCLC cases obtained by TBB with small-diameter GS (retrospective registration) (same number of cases as those actually biopsied with large diameter GS)

[Setting basis]

With regard to the number of tumor cells in specimens, which is the primary endpoint of this study, there have been no reports on the number of tumor cells in specimens obtained by using large-diameter and small-diameter GS. The average number of tumor cells per preparation of two cases of large-diameter GS in our institution was 1410 and 302, and the average number of cells per preparation of three cases of small-diameter GS was 192, 675 and 407. The mean difference between the two groups was 431 (SD: 855). The sample size was calculated assuming that the mean difference between the two groups was 431, with an alpha level set at 0.2 (two-sided) and detection power of 80%. The minimum sample size was calculated as 36 for each group. Considering that cases that required to change the thinner bronchoscope, whose pathological diagnosis other than NSCLC and deviation, the target number of patients for prospective enrollment in the study was set at 80. When 36 NSCLC cases are obtained from specimens collected with large-diameter GS, new enrollment in the study will be terminated. The number of retrospective enrollment cases in the small-diameter GS was equal to the number of prospective enrollment cases, calculated using EZR software version 1.38.

**12.3** Handling of cases

In principle, the research office and the principal investigators will decide on the handling of enrolled cases after consultation with the investigators. If a new problem arises, case handling will be decided by the secretariat and the principal investigator after consultation.

**12.4** Data Handling

If a question arises regarding the handling of data during data compilation and analysis, the principal investigator and the principal investigator will consult with each other before making a decision.

**12.5** Statistical Analysis Items and Analysis Plan

All patients will be analyzed after bronchoscopy is completed and the data are fixed. For all efficacy evaluations, analysis in the largest analysis set (FAS) will be the primary analysis, and analysis in the analysis set (PPS) consistent with the study protocol will be performed as a reference.

12.5.1 Tabulation of research subject background

The distribution and summary statistics of the study subject background data in each analytic population will be calculated for each group.

For the nominal variables, the frequency and percentage of categories are shown for each group.

For continuous variables, summary statistics (number of examples, mean, standard deviation, minimum, median, and maximum) are calculated for each group.

When the number of cells with an expected frequency of less than 5 is 20% or more, Fisher's direct probability calculation method is used, and the t-test is used for continuous variables. The significance level is 5% on both sides.

12.5.2 Effectiveness Analysis

12.5.2.1 Main analysis

The number of tumor cells in specimens obtained with large and small GS will be analyzed using a t-test. The significance level is 5% on both sides.

12.5.2.2 Secondary analysis

Analyses of secondary efficacy endpoints will be conducted for the purpose of providing insights that complement the results of the main analysis. The significance level of the hypothesis test is set at 5% for both sides.

12.5.3 Safety Analysis

Investigate the incidence of complications such as hemorrhage and pneumothorax in patients by TBB with large-diameter and small-diameter GS.

12.5.4 Interim analysis

Interim analysis is not performed in this study.

**12.6** Independent Data Monitoring Committee

An independent data monitoring committee will not be established.

**12.7** Final Analysis

After all bronchoscopy and pathology examinations are completed, data are obtained and cases are fixed for analysis.

# **13．Changes to the study protocol, case report form or analysis plan**

**13.1.** Revisions to the Study Protocol and Case Report Form

When revising the study protocol and case report form, the following procedure should be followed.

1. The principal investigator will promptly submit the revised study protocol and the revised case report form to the head of the institution and obtain approval from the research ethics review committee via the head of the institution.

2. The same procedure will be used to modify the study protocol and case report forms when the direction of the head of the institution, based on the opinion of the research ethics review committee, is acceptable to the principal investigator and the study coordination committee.

**13.2.** Changes to the Statistical Analysis Plan

If the statistical analyst changes the statistical analysis plan, he or she will include all changes in the statistical analysis report for the study. Any changes to the statistical analysis plan will be recorded in the history of the study.

# **14．Data Management**

**14.1** Preparation of the Case Report Form (CRF)

The principal investigator or research co-investigator will prepare a case report form (CRF) for each research subject.

When correcting a CRF once entered, the content and date of the correction must be written with a double line so that the original entry can be read, and the signature or seal of the person correcting must be left behind. In addition, if the correction is not a minor correction such as correction of errors, the reason for the correction should also be written.

The principal investigator signs the CRF after checking the prepared CRF for errors.

The original CRF will be kept in the Research Office.

**14.2** Identification of material that is directly included in the case report form and is the source material (original data).

In this study, the following documents and others will be used as the source documents (original data).

1. Records related to the consent of research subjects and the provision of information to research subjects

Medical records, nursing records, clinical laboratory data and imaging films, bronchoscopy reports and other records that are the basis for preparing case reports. The data stored in the electronic medical record will also be considered as source documents.

2. Documents or records related to the test that are necessary for guidelines related to this test

# **15．Quality Assurance for Clinical Trials**

**15.1** Quality Control

Monitoring will be carried out for the purpose of periodically checking that the study is being conducted safely and in accordance with the protocol and that data are being accurately collected.

15.1.1 Designation of Monitoring Officer and Monitor

The principal investigator will designate a principal monitoring officer and a monitor for the study. The principal monitoring officer and monitors will be nominated from those who have a history of education on the regulatory requirements such as the "Ethical Guidelines for Clinical Research Involving Medical Research Involving Human Subjects" and who fully understand the contents of the study protocol, the research subjects' consent documents and the monitoring protocol of this research.

15.1.2 Monitoring

15.1.2.1 Monitoring of cases

The monitors will have direct access to the original documents (consent forms, medical records, case report forms, etc.) at the Kobe University Hospital during the study.

15.1.2.2 Non-case monitoring

No non-case monitoring will be performed.

**15.2.** Quality Assurance

As this is an exploratory study conducted in a small number of cases, we do not plan to conduct an audit.

**16．Correspondence to the Ethical Guidelines and Declaration of Helsinki**

The study will be conducted in compliance with the Declaration of Helsinki, the Ethical Guidelines for Medical Research Involving Human Subjects, and the Conflict of Interest Management Guidelines of Kobe University.

**17．Consideration for the human rights and safety and disadvantage of research subjects**

**17.1** Consideration for Human Rights (Protection of Personal Information)

The principal investigator or research co-investigator shall comply with the principle of protecting the rights of research subjects against invasion of privacy. They must make every effort to protect the personal information and privacy of research subjects and must not divulge any personal information obtained in the course of the study without just cause. This applies even after the person concerned has left the position. The study will use a list of research subject identification numbers to link the study database and study-related documents to the original data of the research subjects. Limited research subject information, such as gender and date of birth, may be used to identify research subjects and to verify the accuracy of the research subject identification number list, within the limits of all applicable laws and regulations.

The principal investigator or co-investigators will delete any description that could identify an individual (name, initials, address, telephone number, medical record number, etc.) and make the data anonymous. Case study subject identification numbers will be used when registering cases and preparing case report forms. The principal investigator will store and manage the list of subject identification numbers when anonymizing the list of subject identification numbers in accordance with 20. The list of research subject identification numbers will not be made available to the outside world.

When principal investigators disclose information obtained in research, they must be careful not to identify research subjects.

**17.2** Burden on research subjects, anticipated risks (including possible adverse events)

17.2.1 Projected benefits

It is expected that the collection of specimens using the large-diameter GS in this study will result in the benefit of a higher number of tumor cells obtained. In addition, the results of this study may contribute to the advancement of future bronchoscopy methods.

17.2.2 Anticipated Disadvantages (Burden and Risk)

The study population in this study originally required bronchoscopy with EBUS-GS for the diagnosis of peripheral pulmonary lesions in daily practice and is unlikely to be at significant risk of complications beyond those of bronchoscopy in daily practice. In the prospective registration group, change to small-diameter bronchoscopy due to difficulties in obtaining specimens with a large-diameter GS may result in slightly longer examination times and swelling of the tracheal mucosa. Change to thinner bronchoscope should be done as quickly as possible.

On the other hand, no particular disadvantage arises for retrospectively enrolled patients.

**17.3** Consideration of safety and disadvantage

The "inclusion criteria" and "exclusion criteria" have been carefully reviewed to minimize the risks and disadvantages of adverse events incurred in this study. Adverse events that occur will be monitored to ensure that they are within the expected range, and any serious or unanticipated adverse events will be carefully considered and reviewed, and any necessary actions will be taken. Because this study will be conducted within the scope of insurance coverage, no special compensation will be provided and complications will be covered by insurance when they occur. In the event of a serious adverse event, the attending physician will provide honest medical care to the subject.

As a general rule, the cost of treatment provided for the health hazards of the study will be paid for by health insurance and patient contributions.

**17.4** Report to the head of the research institute

The principal investigator shall report the following in writing to the head of the research organization. The principal investigator shall report on the following in writing to the head of the institution.

1. Research Progress

2. Facts or information that undermine the ethical validity or scientific rationality of the research, or information that may undermine the ethical validity or scientific rationality of the research and that may affect the continuation of the research.

3. When a fact or information is obtained that undermines or may undermine the appropriateness of the conduct of the research or the reliability of the results of the research

4. When the research is completed (stopped or discontinued)

5. When a serious concern arises from the perspective of respecting the human rights of research subjects, etc., or from the perspective of conducting research, such as the disclosure of information related to the research

6. Others

**17.5** Approaches to Disclosure of Information on Genetic Mutations and Incidental Findings

It is unlikely that this study will yield any important findings on genetic characteristics.

**17.6** Genetic counseling

This study is not subject to the Ethical Guidelines for Human Genome and Genetic Studies and therefore genetic counseling will not be conducted.

**17.7** Secondary use of data and samples

Data on research subjects obtained in this study will be compiled into a database and may be used in other studies in the future. When conducting other research, a new research plan will be developed and approved by the Ethics Review Committee. In such cases, written consent will not be sought from individual research subjects, but research-related information will be disclosed in an appropriate manner.

**18．Approach to explain to research subjects and obtain their consent (informed consent)**

**18.1** Preparation and revision of consent and other explanatory documents

The principal investigator or co-investigator will prepare the consent document and other explanatory documents used to obtain consent to participate in the study from research subjects in as easy explanation as possible. They will also revise the consent document and other explanatory documents when deemed necessary. The principal investigator or principal investigator will give the potential research subjects an opportunity to ask questions and sufficient time to decide whether to consent, and will obtain their voluntary consent after confirming that they have a good understanding of the study.

The principal investigator or principal investigator will submit the prepared or revised consent narrative document and other explanatory documents to the Intervention Research Ethics Review Board for approval.

**18.2** Procedures for receiving informed consent from a proxy

The prospective enrollment group in this study must obtain consent from the study subjects themselves.

For the backward-looking enrollment group, it is not possible to obtain informed consent from individual research subjects, because some deaths and other unreachable cases will be included. Therefore, a release of information document will be made available on the Internet to allow subjects the opportunity to refuse consent to be enrolled in the study.

**18.3** Procedures for receiving an informed assent

Not applicable.

**18.4** Explanatory notes to research subjects and their proxies

The narrative document prepared by the principal investigator shall include the following

1. Introduction (that this study involves research)

2. the purpose of this clinical trial

3. Reasons for asking you to participate in the study

4. The method (treatment) and duration of this clinical trial

5. The number of participants in this test

6. What to do after the completion of clinical trials

7. Expected Effects

8. Expected complications and risks

9. if your health is harmed during this test

10. Disclosure of information such as genetic analysis and expected and anticipated benefits and disadvantages

11. Fees and rewards for this study

12. About this and other examination

13. not to be disadvantaged if you do not agree

14. Withdrawal after consent

15.How to store and use samples, etc. and storage period

16. Disclosure of treatment plans and other information

17. Protection of Privacy

18. Access to medical records, etc.

19. Ownership of intellectual property rights

20. sources of funding and conflicts of interest related to this research

21. What you need to protect

22. Contact for inquiries

23. Research Institutions and Principal Investigators

**19．The cost of the research subjects**

**19.1** Compensation for Health Damage

This clinical research will use drugs and medical devices that have been approved by the Ministry of Health, Labour and Welfare. Therefore, if any damage to health occurs as a result of treatment in this clinical research, the principal investigator or research co-investigator will be responsible for the treatment, and no financial compensation will be provided. If a disorder occurs, appropriate treatment and therapy will be provided as soon as possible, but the costs of such treatment will be borne by the research subjects.

**19.2** Overview of cost sharing

Because the tests and medications used in this study will be administered within the normal course of insurance, there will be no cost to the study subjects for participating in the study.

**20．Retention of documents and records**

In accordance with the "Guidelines on the Retention Period of Research Data at the Kobe University Graduate School of Medicine", the principal investigator must ensure that all important documents related to the conduct of the research (copies of application documents to the Ethical Review Committee, letters of notification from the head of the research institution, copies of applications and reports, information disclosure documents, data revision history, etc.) are kept on file in accordance with the Guidelines. (e.g., documents or records that support information used in the research, such as entries in a notebook) must be stored in a lockable location for 10 years after the cessation or completion of the research or for 10 years from the date of publication of the results of the research (e.g., a paper or other research result), whichever is later, and then disposed of in a manner that does not allow for identification of individuals.

The samples (specimens) pertaining to this study will be retained for five years (in principle) from the date when the final publication of the results of this study is reported, and then disposed of in a manner that does not allow identification of individuals.

**21．Registration of the research plan**

The study was based on the recommendations of the International Committee of Medical Journal Editors (ICMJE) and was conducted on the University hospital Medical Information Network Clinical Trials Registry System (University hospital Medical Register your study abstract with the Information Network-Clinical Trial Registry (UMIN-CTR) (Registration number: 000032599). Update the abstract as needed, such as when the research protocol is changed.

**22．Disclosure of research funding sources and COI status of each researcher**

**22.1** Sources of funding for research

Because this study will be conducted within the scope of insurance coverage, no funds are required for the examination and treatment of the study itself. The cost of conference presentations will be covered by the research funds of the Department of Respiratory Medicine, Department of Internal Medicine, Graduate School of Medicine, Kobe University.

**22.2** Conflict of Interest in Research

There are no conflicts of interest to be disclosed regarding this study.

**23．Attribution of Research Results and Publication of Research Results**

The results, data and intellectual property rights obtained from this study belong to the Department of Respiratory Medicine, Kobe University Hospital. The specific handling and distribution of the data will be decided through discussion.

The results of the study will be presented at an academic conference and then submitted as a paper in an English language journal. Presentations at domestic conferences will also be made as necessary. In principle, the principal author of the publication of the research results should be the principal investigator, followed by the principal investigator. Coauthors of the papers below this level shall be selected in accordance with the restrictions on the submission of papers.

All co-authors must have reviewed the content of the paper and agreed on the content of the publication before submission. The principal investigator may exclude a researcher from the list of co-authors with the approval of the principal investigator if there is no agreement on the content of the paper after discussion. Since the conference presentation may involve more than one presentation, presentations shall be made on a rotating basis by those appointed by the research secretariat and the principal investigator.

# **24． Research Organization**

# **24.1** Research institutions

1. Principal Investigators and Research Associates

（Name）

○Motoko Tachihara （○: Principal Investigator）

Yoshihiro Nishimura

Kazuyuki Kobayashi

Masatsugu Yamamoto

Naoko Katsurada

Naoe Jimbo

Tatsunori Kiriu

Yuichiro Yasuda

Takehiro Otoshi

Asuka Yoshizaki

　 Kiyoko Koyama

Masako Yumura

Koichi Furukawa

　 Jun-ya Yoshioka

Chihiro Mimura

Hiroki Sato

Naoya Takata

2. Research Secretariat

Naoko Katsurada, Assistant Professor, Department of Respiratory Medicine, Department of Internal Medicine, Kobe University Graduate School of Medicine, Kobe, Japan

7-5-2 Kusunomachi, Chuo-ku, Kobe 650-0017, Japan

Tel: +81-78-382-5660

FAX: +81-78-382-5661

E-mail: nk1208@med.kobe-u.ac.jp

3．Data Management Officer

Naoko Katsurada, Assistant Professor, Department of Respiratory Medicine, Department of Internal Medicine, Kobe University Graduate School of Medicine, Kobe, Japan

4．Responsible for Monitoring

Tatsuya Nagano, Assistant Professor, Department of Respiratory Medicine, Graduate School of Medicine

5．Responsible for the audit

None (no audit)

6．Head of Statistical Analysis

Naoko Katsurada, Assistant Professor, Department of Respiratory Medicine, Department of Internal Medicine, Kobe University Graduate School of Medicine, Kobe, Japan

**24.3** Consignment Work and Consignee

No commissioned work

# **25．References**

1. National Cancer Institute Cancer Information Service Latest Cancer Statistics <https://ganjoho.jp/reg_stat/statistics/stat/summary.html>

2. The Japanese Lung Cancer Society Guide to PD-L1 Testing in Lung Cancer Patients, Version 1

https://www.haigan.gr.jp/uploads/photos/1400.pdf

3. Hayama M, Izumo T, Matsumoto Y, Chavez C, Tsuchida T, Sasada S. Complications with Endobronchial Ultrasound with a Guide Sheath for the Diagnosis of Peripheral Pulmonary Lesions. Respiration; international review of thoracic diseases. 2015; 90:129-35.

4. Minami D, Takigawa N, Morichika D, Kubo T, Ohashi K, Sato A, Hotta K, Tabata M, Tanimoto M, Kiura K. Endobronchial ultrasound-guided transbronchial biopsy with or without a guide sheath for diagnosis of lung cancer. Respiratory investigation. 2015; 53:93-7.

5. Ishida T, Asano F, Yamazaki K, Shinagawa N, Oizumi S, Moriya H, Munakata M, Nishimura M; Virtual Navigation in Japan Trial Group. Virtual bronchoscopic navigation combined with endobronchial ultrasound to diagnose small peripheral pulmonary lesions: a randomised trial. Thorax. 2011; 66:1072-7.

6. Zhang L, Wu H, Wang G. Endobronchial ultrasonography using a guide sheath technique for diagnosis of peripheral pulmonary lesions. Endoscopic ultrasound. 2017; 6:292-9.

7. Xu CH, Yuan Q, Yu LK, Wang W, Lin Y. Endobronchial ultrasound transbronchial biopsy with guide-sheath for the diagnosis of solitary pulmonary nodules. Oncotarget. 2017; 8:58272-7.

8. Chavez C, Sasada S, Izumo T, Watanabe J, Katsurada M, Matsumoto Y, Tsuchida T. Endobronchial ultrasound with a guide sheath for small malignant pulmonary nodules: a retrospective comparison between central and peripheral locations. Journal of thoracic disease. 2015; 7:596-602.

revision history

| version number | version number | Reasons for revision/content |
| --- | --- | --- |
| 1st ed. | May 1, 2018 | newly enacted |
| 1.1 | July 18, 2018 | Revised (Setting of target number of cases, and addition of main endpoints) |
| 1.2 | September 18, 2018 | Revised (revised selection criteria and case enrollment) |
| 1.3 | March 31, 2019 | Revision (Change of Research Staff) |
| 1.4 | June 12, 2019 | Revision (Setting of target number of cases, change in researcher) |
| 1.5 | October 21, 2019 | Revision (Extension of test period) |
| 1.6 | March 31, 2020 | Revision (Change of Research Staff) |
